# CD226 Attenuates Treg Proliferation via Akt and Erk Signaling in an EAE Model

**DOI:** 10.3389/fimmu.2020.01883

**Published:** 2020-08-21

**Authors:** Ning Wang, Hongyu Yi, Liang Fang, Jingyi Jin, Qianli Ma, Yuting Shen, Juan Li, Shuang Liang, Jie Xiong, Zhuo Li, Hanyu Zeng, Fengliang Jiang, Boquan Jin, Lihua Chen

**Affiliations:** ^1^Department of Immunology, The Fourth Military Medical University, Xi'an, China; ^2^Department of Immunology, Xi'an Medical University, Xi'an, China

**Keywords:** CD226, EAE, Tregs, proliferation, suppression, Akt signaling, Erk signaling

## Abstract

Cluster of differentiation 226 (CD226) molecules play a crucial role in the activation of effector CD4^+^ T cells during the immune response process, but a cell-intrinsic function of CD226 in CD4^+^ T subsets is not clear. In this study, we showed that *Cd226*^−/−^ mice were resistant to myelin oligodendrocyte glycoprotein peptide 35-55 (MOG_35−55_)-induced experimental autoimmune encephalomyelitis (EAE) with highly expressed IL-10^+^CD4^+^ T cells and downregulated IL-17A^+^CD4^+^ T cells when compared with wild-type (WT) mice. Th17 cell infiltration into the central nervous system (CNS) was largely decreased in the absence of CD226 during EAE. CD226 deficiency facilitated the proliferation of regulatory T cells (Tregs), with increased numbers of Tregs observed in EAE mice, and supported the elevated induced regulatory T cell (iTregs) proliferation *in vitro*. The Akt and Erk signaling pathways were shown to be involved in *Cd226*^−/−^ Treg proliferation and function *in vivo* and *in vitro*. These findings collectively indicate that CD226 is a key molecule regulating the Treg-mediated suppression of autoimmune responses by inhibiting Treg proliferation. Thus, the results of this study identify additional mechanisms by which CD226 regulates Treg functions in EAE and supports the potential therapeutic effects of anti-CD226 molecules on autoimmune diseases.

## Introduction

Cluster of differentiation 226 (CD226) is an adhesion molecule belonging to the immunoglobulin superfamily (1) that is ubiquitously and constitutively expressed on the majority of natural killer (NK) cells, T cells, and other immune cells and competes with the immunoreceptor tyrosine inhibition motif domain (TIGIT) for binding to the ligands CD155 and CD112 (2). Previous studies have reported that CD226 triggers the effector function of NK cells and contributes to Lymphocyte Function-associated Antigen (LFA)-1 signaling that promotes T cell activation (3). As an adhesion molecule, CD226 mediates cellular adhesion during pathological inflammation, and accumulating reports have shown that CD226 is associated with several autoimmune diseases (4, 5). Moreover, CD226 has been identified as an autoimmune susceptibility gene, and its allelic variants are linked to the development of multiple sclerosis (MS), systemic lupus erythaematosus (SLE), rheumatoid arthritis (RA), and juvenile idiopathic arthritis (JIA) (6, 7). Additionally, CD226 has also been demonstrated to be involved in the pathogenesis of autoimmune diseases by promoting self-reactive CD4^+^ T cell activation (8, 9) and triggering naïve CD4^+^ T cell differentiation and proliferation (3). Although the role of CD226 in autoimmune diseases has been well-documented, the molecular mechanisms associated with CD226 activity in these diseases are not clear.

MS is a chronic demyelinating autoimmune disease of the central nervous system (CNS) that is primarily mediated by T lymphocytes and is characterized by widespread inflammatory processes in the CNS, immune dysregulation, and immune over-activity. However, there are limited effective treatment solutions for this disease (10). The experimental autoimmune encephalomyelitis (EAE) model is one of the most suitable models for studying the pathogenesis of MS (11). Studies have shown that CD4^+^ T cells from T cell receptor (TCR) transgenic mice engineered to express TCR-specific myelin oligodendrocyte glycoprotein peptide 35-55 (MOG_35−55_) develop CNS inflammation, suggesting that CD4^+^ T cells play a central role in EAE (12). Previous studies have shown that excessive activation of auto-reactive CD4^+^ T cells, especially helper T (Th) 17 cells and the weakened function of regulatory T cells (Tregs) facilitates the development and advancement of MS (13). Although some studies have preliminarily confirmed that the reduction of the suppressive capabilities and the altered molecular expression of Tregs is linked to the pathogenesis of MS, the exact mechanism of auto-reactive CD4^+^ T cell dysregulation remains unclear (14, 15).

Tregs are characterized by the expression of transcription factor Foxp3 and play a crucial role in maintaining peripheral immune tolerance during autoimmune disease (16). Many investigations have reported that Tregs inhibit autoimmune responses by suppressing the effector CD4^+^ T cell (T_eff_) subsets (17). Under the appropriate conditions, such as a specific cytokine environment, Tregs inhibit Th17 cell differentiation via repression of the transcription factor RORγt (18). However, under inflammatory conditions, some Tregs may lose Foxp3 expression and lineage stability, which may contribute to uncontrolled inflammation (19). It has been reported that CD226 opposes TIGIT to disrupt Tregs in melanoma (20). However, the role of CD226 in modulating the functions of Tregs in EAE remains unclear.

To better elucidate the role of CD226 in CD4^+^ T subsets, especially in Tregs, we generated CD226 knockout (*Cd226*^−/−^) mice and assessed the role of this protein in CD4^+^ Foxp3^−^ traditional T (Tconv) cells and CD4^+^ CD25^+^ Foxp3^+^ Tregs during EAE pathogenesis. We observed that Tconv cells and Tregs from EAE mouse model spleens showed differential expression of CD226 and that *Cd226*^−/−^ mice were more resistant to EAE than wild-type (WT) mice. Thus, we speculated that the CD226 deletion inhibits the pathological immune response in the EAE model. After comparing the number of Tregs and Th17 cells in the CNS and spleens of mice, we observed the presence of more Tregs but fewer Th17 cells in the spleens, and Th17 cell infiltration into the CNS was decreased in the absence of CD226 during EAE. To further assess whether CD226 is associated with Treg functions, we sorted Tregs from splenocytes and collected *in vitro*-derived induced regulatory T cells (iTregs) to assess their proliferation and suppressive capacity. We demonstrated that CD226 deficiency facilitates the Treg cell-mediated suppression of autoimmune responses in EAE mice. Then, we sorted Tregs from splenocytes, generated iTregs *in vitro* and observed that the Akt and Erk signaling pathways are involved in *Cd226*^−/−^ Treg proliferation and function. These findings collectively indicate that CD226 is a key molecule in the Treg-mediated suppression of autoimmune responses because it inhibits Treg proliferation. Thus, we provide further mechanisms by which CD226 molecules are involved in Treg functions in EAE and evidence for the potential therapeutic effect of targeting CD226 in autoimmune diseases.

## Materials and Methods

### Mice

WT C57BL/6 mice were purchased from the Nanjing Biomedical Research Institute of Nanjing University (Nanjing, China). CD226 knockout (*Cd226*^−/−^) mice in a C57BL/6 background were a kind gift from Professor Marco Colonna. To generate homozygous *Cd226*^−/−^ mice, the *Cd226*^−/−^ mice were back-crossed to C57BL/6 mice (8 weeks) and then propagated by *Cd226*^+/−^ × *Cd226*^+/−^ mating. The WT (*Cd226*^+/+^) mice used as controls in our experiments were the littermates of the *Cd226*^−/−^ mice. All the mice were bred under specific pathogen-free (SPF) conditions at the Experimental Animals Center of the Fourth Military Medical University. All experiments were approved by the Scientific Investigation Board of the Fourth Military Medical University, Xi'an (permit number XJYYLL-2014433). All animals were treated according to the Guide for the Care and Use of Laboratory Animals (NIH, Bethesda, USA).

### Induction and Assessment of the EAE Model

To induce EAE, mice were subcutaneously immunized in their flanks with 200 μg of MOG_35−55_ (purity >98%, GT10451-0614, Bankpeptide Biotec, China) in complete Freund's adjuvant (CFA; F-5881, Sigma, St. Louis, MO, USA) containing 4 mg of a thermally inactivated tuberculosis strain (2240273, Difco, Lawrence, USA). On days 0 and 2, the immunized mice were intraperitoneally injected with 200 ng of Bordetella pertussis toxin (P2980, Sigma, St. Louis, MO, USA) (21). The mice were monitored and weighed daily. The clinical score of mouse EAE was determined according to the following judging criteria: 0, no symptom and disease; 1, limp tail; 2, hind-limb weakness; 3, partial hind-limb paralysis; 4, complete paralysis of one or more limbs; and 5, moribund state or dead (21).

### Cell Isolation and Culture

Fifteen to eighteen days after immunization, the spleens were removed, and Tregs were isolated using a CD4^+^CD25^+^ regulatory T cell isolation kit (130-091-041, MACS Miltenyi Biotec, Germany) following the manufacturer's instructions. To sort purified CD4^+^ T cells using magnetic beads, erythrocyte-depleted splenocyte suspensions from *Cd226*^−/−^ or WT mice were negatively selected using the Mojosort™ Mouse CD4 T Cell Isolation kit (480006, Biolegend, San Diego, CA, USA). The purified CD4^+^ T cells (cell purity >95%) were cultured in RPMI 1640 medium and stimulated with plate-bound anti-CD3 (3 μg/ml, LEAF™ Purified anti-mouse CD3ε, 100313, Biolegend, San Diego, CA, USA) and soluble anti-CD28 (5 μg/ml, LEAF™ Purified anti-mouse CD28, 102111, Biolegend, San Diego, CA, USA) in the presence of recombinant murine IL-2 (2 ng/ml, 212-12, Peprotech, Cranbury, NJ, USA).

To prepare the purified naïve CD4^+^ T cells, erythrocyte-depleted splenocyte suspensions from *Cd226*^−/−^ or WT mice were negatively selected using a Mojosort™ Mouse CD4 Naïve T Cell Isolation kit (480039, Biolegend, San Diego, CA, USA). The purity of naïve CD4^+^ T cells was >95%. A 96-well plate was coated with 3 μg/ml of anti-CD3 antibody (100313, Biolegend, San Diego, CA, USA) in PBS at 4°C overnight. Subsequently, soluble anti-CD28 (5 μg/ml, 102111, Biolegend, San Diego, CA, USA) and recombinant TGF-β1 (5 ng/ml, 100-21, Peprotech, Cranbury, NJ, USA) were added, and the cells were incubated for 3 days to polarize the iTregs (21).

### CFSE Labeling and iTreg Inhibition Assay

To determine the iTreg proliferation ability, iTregs were labeled for 8 min at 37°C with 2.5 μM carboxyfluorescein diacetate succinimidyl ester (CFSE) according to the manufacturer's protocol (423801, Biolegend, San Diego, CA, USA). Then, the iTregs were washed three times with RPMI 1640 medium and cultured with plated-bound anti-CD3 (5 μg/ml, 100302, Biolegend, San Diego, CA, USA) and soluble anti-CD28 (5 μg/ml, 102111, Biolegend, San Diego, CA, USA) in RPMI 1640 complete medium with 10% FBS for 3 days. To determine the inhibitory activity of the iTregs, immunogenic bead-sorted CD4^+^ T cells labeled with CFSE were used as responders. The CD4^+^ T cells were cultured in 96-well plates with FACS-sorted CD4^+^CD25^+^ iTregs for 5 days at various ratios in medium supplemented with soluble anti-CD3 (5 μg/ml) and anti-CD28 (5 μg/ml).

### CNS Tissue Isolation and Histology Assay

Mouse lumbar spinal cords and brains tissue were removed at the peak of EAE after cardiac perfusion with PBS and fixed in 4% paraformaldehyde (PFA; P-6148, Sigma, St. Louis, MO, USA). Fixed tissues were washed with ethanol, embedded in paraffin, and used to prepare 20-μm cross-sections that were stained with Luxol fast blue (LFB) to visualize demyelination and H&E to assess cellular infiltration. Spinal cords were used to prepare 20-μm longitudinal sections or cross-sections for immunohistochemical staining for histopathological assessments. All images were captured using an Olympus microscope (Olympus Corporation, Tokyo, Japan), and quantification was performed using ImageJ Pro (Rawak Software Inc., Stuttgart, Germany). For immunofluorescence assays, CNS tissues were fixed in 4% PFA for 10 h, rehydrated in a 30% sucrose solution for 24 h and frozen in OCT for cross-sectioning. Brain tissues were blocked with H_2_O_2_ and then stained with anti-Foxp3 (bsm-52079R, Bioss, China) or anti-RORγt (bs-23110R, Bioss, China) antibodies at 4°C overnight. On the second day, The Alexa Fluor 488 conjugated Goat anti-Rabbit (ab150081, abcam, USA) as secondary antibodies were used following the manufacturer's instructions. All images were captured using a Zeiss 710 LSM confocal microscope (Carl Zeiss Inc., Germany). RORγt^+^ or Foxp3^+^ cells were quantified as the percentage of the total cells stained with DAPI in each section. All slides were blinded during the assessment, and at least four non-consecutive sections of the lumbar spinal cord or brain of each animal were evaluated by two individuals. The number of animals analyzed and used in the experiment are indicated in the figure legends.

### Isolation of Mononuclear Cells in the Spinal Cord and Brain

The spinal cords and brains of mice were removed at the peak of EAE after cardiac perfusion and washed twice with fresh pre-cooled PBS. After homogenization and collection of these tissues, the CNS mononuclear cells were harvested by centrifugation with 70–30% Percoll gradients (28-9038-34, GE Healthcare, Sweden), passed through a 70-μm filter and then washed with RPMI 1640 medium according to the manufacturer's protocol. The viability of the cells was detected using trypan blue staining (22).

### Flow Cytometry

The expression of cell surface molecules was detected by staining with antibodies against CD4 (Percp anti-mouse CD4, 100538, Biolegend, San Diego, CA, USA), CD69 (FITC anti-mouse CD69, 104505, Biolegend, San Diego, CA, USA), CD25 (PE anti-mouse CD25, 102007, Biolegend, San Diego, CA, USA), CD226 (APC anti-mouse CD226, 125509, Biolegend, San Diego, CA, USA), and TIGIT (APC anti-mouse TIGIT, 131510, Biolegend, San Diego, CA, USA), diluting each antibody according to the manufacturer's instructions. The *in vitro* apoptosis of iTregs after polarization from naïve CD4^+^ T cells was determined using a FITC Annexin V Apoptosis Detection kit with propidium iodide (PI; 640914 Biolegend, San Diego, CA, USA).

To examine the intracellular expression of the cytokines IFN-γ (PE anti-mouse IFN-γ, 505807, Biolegend, San Diego, CA, USA), IL-4 (PE anti-mouse IL-4, 504104, Biolegend, San Diego, CA, USA), IL-10 (PE anti-mouse IL-10, 505008, Biolegend, San Diego, CA, USA), and IL-17A (PE anti-mouse IL-17A, 506904, Biolegend, San Diego, CA, USA), the cells were stimulated with Cell Activation Cocktail (with Brefeldin A) (423303, Biolegend, San Diego, CA, USA) for 6 h according to the manufacturer's protocols. To determine the amount of Foxp3^+^ and Ki67^+^ cells in the population, the cells were sequentially fixed, permeabilized (Fixation/permeabilization Diluent, 00-5223, eBioscience, San Diego, CA, USA) and stained with Foxp3 (Alexa Fluor 488 anti-mouse FOXP3, 320011, Biolegend, San Diego, CA, USA) or Ki67 (PE anti-mouse Ki67, 652403, Biolegend, San Diego, CA, USA).

### Quantitative Reverse Transcriptase-Polymerase Chain Reaction (RT-qPCR)

RNA was isolated with RNAiso Plus (9109, TaKaRa, Japan) according to the manufacturer's protocol. The cDNA was synthesized with PrimeScript RT Master Mix (RRO36A, TaKaRa, Japan), and PCR was performed using SYBR PremixEx Taq™ II (RR820A, TaKaRa, Japan). The sequences of primers used for Tregs and Th17 cell-related molecules are listed in Supplemental Table 1. The primers were purchased from Applied Biosystems (AUGCT, China). The samples were amplified over 40 cycles using the following thermocycling program: 15 s at 95°C and 1 min at 60°C. GAPDH gene expression was used as an endogenous reference to calculate relative mRNA expression.

### Western Blotting

A total of 5 × 10^6^ isolated cells or induced iTregs were stimulated with anti-CD3 (5 μg/ml)/anti-CD28 (5 μg/ml) plus IL-2 (2 ng/ml) in the presence or absence of TGF-β1 (5 ng/ml) for the indicated times. The cells were harvested and then lysed using RIPA (70166, Sigma, St. Louis, MO, USA) to obtain protein. The protein concentration was determined using a Pierce BCA Protein Assay kit (#23225, Thermo, USA), 15 μl of protein lysate was loaded onto 8% SDS-PAGE gels, and the proteins were blotted onto nitrocellulose membranes. The membranes were blocked with 5% skim milk (70166, Sigma-Aldrich, St. Louis, MO, USA) and incubated at 4°C for 12 h, which was followed by an incubation with horseradish peroxidase-conjugated secondary antibodies. The expression of the indicated proteins was detected with antibodies against the following: Akt (#207, Cell Signaling, USA), phospho-Akt (Ser473) (#207, Cell Signaling, USA), p44/42 MAPK (Erk1/2) (137F5) (#5594, Cell Signaling, USA), phospho-p44/42 (Erk1/2) (#5595, Cell Signaling, USA) and β-actin (#4970, Cell Signaling, USA). The antibodies were diluted 1:2,000 (phospho-antibodies and β-actin antibodies) or 1:1,000 (Akt and Erk1/2 antibodies). Mouse anti-rabbit IgG mAb (HRP conjugate) (#93702, Cell Signaling, USA) was used as a secondary antibody at a 1:2,000 dilution. The protein bands were detected using an ECL detection system and visualized with a Tanon™ High-sig ECL Western Blotting Substrate kit according to the manufacturer's instructions (180-500, Tanon, China).

### Statistical Analysis

Statistical analysis was performed using GraphPad Prism 6 (Prism Software, Lake Forest, CA). For parametric analysis, Student's *t*-test was used. Statistical comparison of EAE severity and body weight between experimental groups was performed using a non-parametric Mann-Whitney *U*-test (two-tailed). The data are presented as the mean ± standard error of the mean (SEM). Differences achieving values of *P* < 0.05 were considered significant, while *P* > 0.05 was considered non-significant (ns). FlowJo V10 (Tree Star, CA, USA) was used to analyse flow cytometry data. The histology assays were performed with Image-Pro software.

## Results

### Tregs Fail to Upregulate CD226 Expression Levels During EAE Conditions

**Figure 1:**
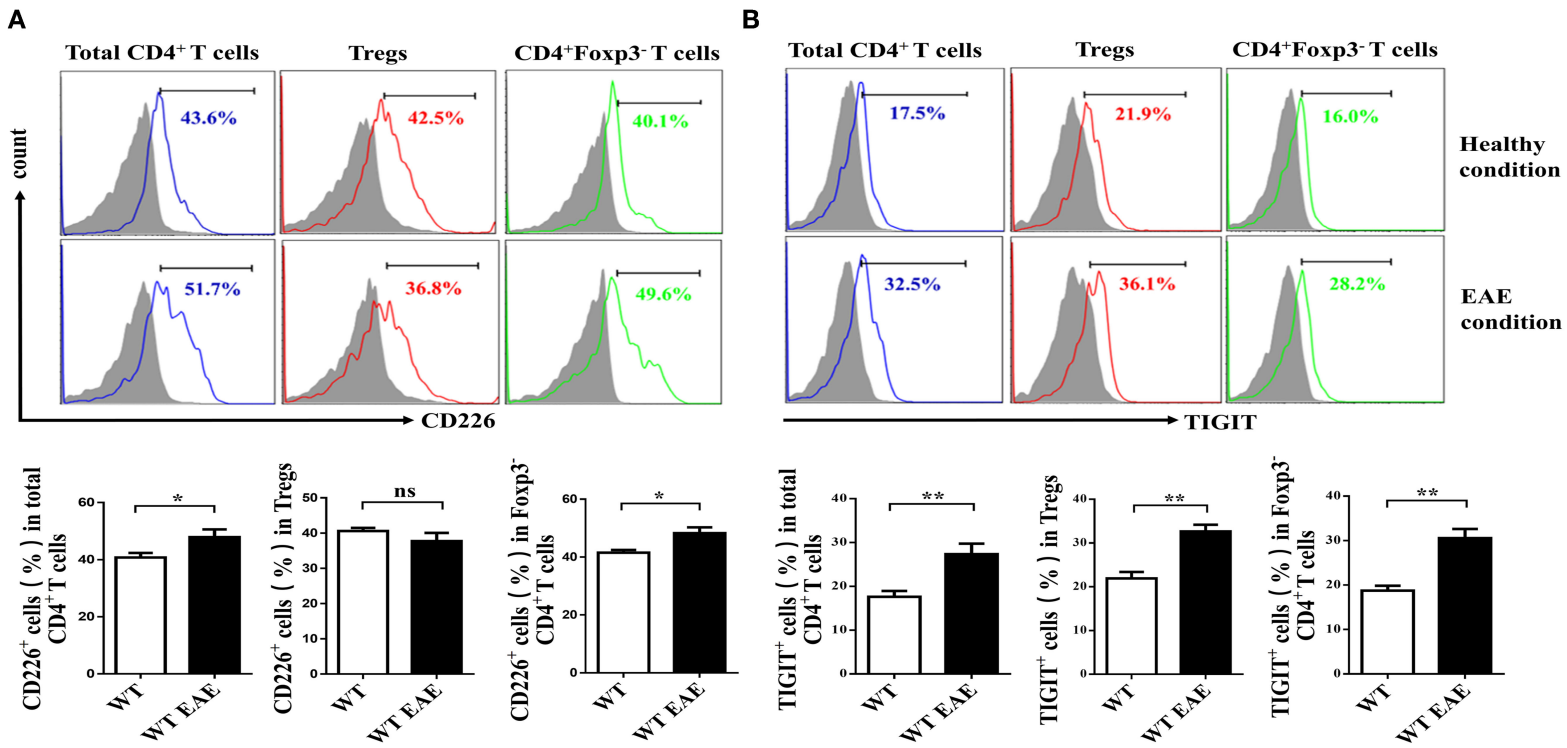
Tregs fail to upregulate CD226 at the peak of EAE. **(A)** The percentage of CD226^+^ cells of the total CD4^+^ T cells, CD4^+^CD25^+^ Foxp3^+^ Tregs or CD4^+^Foxp3^−^ CD4^+^ Tconv cells derived from the spleens of WT and WT EAE mice at the peak of EAE (days 15–18 after MOG_35−55_ immunization) was assessed by flow cytometry (FCM) (*n* = 10). **(B)** The percentage of TIGIT^+^ cells pf the total CD4^+^ T cells, CD4^+^CD25^+^Foxp3^+^ Tregs or CD4^+^Foxp3^−^ T cells derived from the spleens of WT and WT EAE mice (at the peak of EAE) were assessed by FCM (*n* = 10). The results represent five independent experiments **(A,B)**. The data are presented as the mean ± SEM. Frequencies are displayed below the histograms. ^ns^*P* > 0.05, *P < 0.05, **P < 0.01.

CD226 is crucial for the activation of CD4^+^ T cells (23), which contributes to the LFA-1 costimulatory signaling that promotes Th1 cell differentiation (3). To determine whether CD226 is involved in the function of CD4^+^ T cell subsets, we first evaluated the level of CD226 expression of total spleen CD4^+^ T cells, CD4^+^ Foxp3^−^ Tconv cells and CD4^+^CD25^+^Foxp3^+^ Tregs of WT mice. Under healthy conditions, the expression levels of CD226 in total CD4^+^ T cells and their subset cells were comparable. Notably, the level of CD226 expression was upregulated in total CD4^+^ T cells and in Tconv cells under EAE conditions compared with that observed under healthy conditions, in agreement with previous reports that CD226 plays an important role in promoting CD4^+^ T cell activation in response to autoimmune diseases (24, 25). However, mouse spleen Tregs failed to upregulate CD226 under EAE conditions compared with that observed under healthy conditions (Figure 1A). These observations suggest a potential differential role of CD226 in regulating the function of CD4^+^ T cell subsets during healthy and EAE conditions. Because TIGIT and CD226 both bind the ligands CD155 and CD112, we next asked whether TIGIT is also differentially expressed in CD4^+^ T subsets in mice during EAE conditions. The results revealed that CD4^+^ T subsets expressed higher levels of TIGIT during EAE pathogenesis than that observed during healthy conditions. Notably, no differential TIGIT expression was observed among CD4^+^ T cell subsets under healthy or EAE conditions (Figure 1B). Fourcade et al. reported a low CD226/TIGIT ratio in Tregs that was correlated with enhanced Treg stability and function in melanoma (20). Therefore, we speculate that the differential expression of CD226 in CD4^+^ T subsets during EAE can affect the function of Tregs.

### *Cd226*^–/–^ Mice Are Less Susceptible to EAE and Lower Levels of Infiltrated Th17 Cells in the CNS of *Cd226*^–/–^ Mice

**Figure 2:**
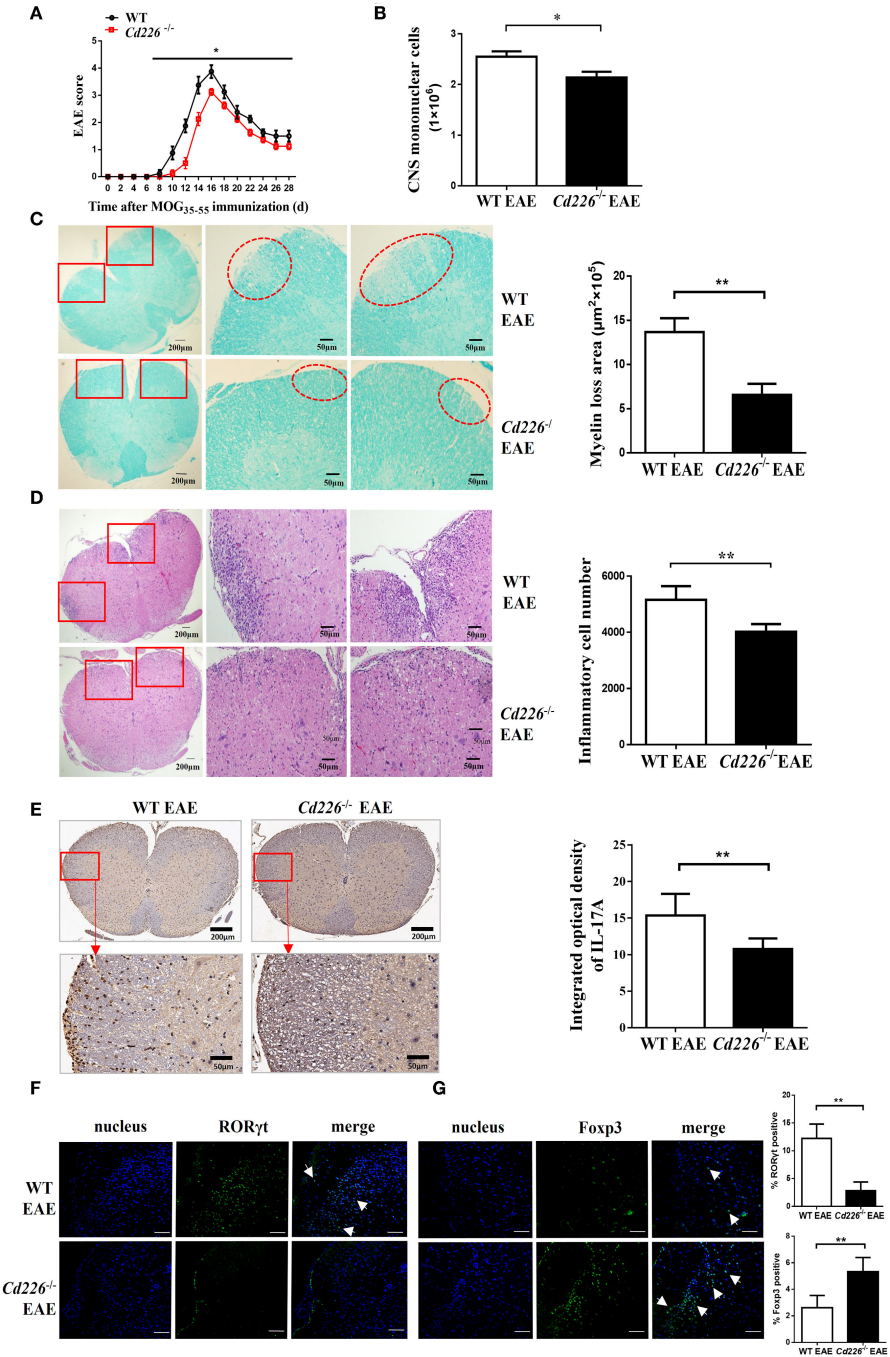
*Cd226*^−/−^ mice are less susceptible to EAE, and lower levels of Th17 cells were infiltrated into the CNS of *Cd226*^−/−^ mice during EAE. **(A)** *Cd226*^−/−^ and WT mice were immunized to induce EAE. The clinical score was monitored at the indicated days after immunization with MOG_35−55_ in CFA (*n* = 16). EAE scores were analyzed by a non-parametric Mann-Whiney *U*-test (two-tailed). The results represent eight independent experiments. **(B)** Mice were killed at the peak of EAE. Total mononuclear cells infiltrated in the CNS of WT and *Cd226*^−/−^ mice were calculated (*n* = 6). **(C)** LFB staining of the lumbar region of the spinal cord (cross-sections) from WT and *Cd226*^−/−^ mice at the peak of EAE (*n* = 6). Demyelination of representative spinal cord sections is indicated by loss of blue staining (scale bars indicate 200 and 50 μm for lower and higher magnification, respectively), and the total demyelinated area was measured with ImageJ. **(D)** Histological analysis of lymphocyte infiltration in the lumbar region of the spinal cord (cross-sections) of the indicated mice by H&E staining (scale bars indicate 200 and 50 μm for lower and higher magnification, respectively) (*n* = 6). **(E)** IL-17A expression in the lumbar region of the spinal cord (cross-sections) at days 15–18 post-immunization was detected by immunohistochemical staining (scale bars indicate 200 and 50 μm for lower and higher magnification, respectively) (*n* = 6). RORγt **(F)** and Foxp3 **(G)** expression in the brains of *Cd226*^−/−^ mice and WT littermates at the peak of EAE were detected by immunofluorescence staining using RORγt (green) or Foxp3 (green) antibodies, respectively. Nuclei are shown in blue (DAPI) (3 cross-sections of brain per mouse, *n* = 6). The white arrowhead indicates Th17 cells or Tregs. Scale bar, 100 μm. The results represent three independent experiments **(B–G)**. The data are presented as the mean ± SEM. ^ns^*P* > 0.05, *P < 0.05, **P < 0.005.

To evaluate the function of CD226 in CD4^+^ T subset cells, we constructed *Cd226*^−/−^ mice with knocked out CD226 (Supplemental Figure 1). We investigated the difference between *Cd226*^−/−^ and WT mice under inflammatory conditions and used the MOG_35−55_-induced EAE model to monitor the pathological effect of CD226. We noticed that EAE could be induced in both *Cd226*^−/−^ and WT mice with 100% incidence, and no mice died in either group during EAE. We then tested the outcome of these two groups during the development of EAE. Our results showed that WT mice progressively developed EAE based on clinical scoring (Figure 2A), with a maximum mean score of 4.1 ± 0.23, while *Cd226*^−/−^ mice showed a delayed onset of EAE (maximum mean score of 3.1 ± 0.1). We also observed that CD226 knockout mice showed suppressed disease progression and attenuated clinical scores induced by MOG_35−55_ (Figure 2A). Moreover, *Cd226*^−/−^ mice tended to show reduced weight loss (days 4–6 and 20–28 after MOG_35−55_ immunization), a feature associated with EAE progression (26), although this effect was not significant (Supplemental Figure 2A). These data collectively indicated that CD226 deficiency in EAE mice may alleviate the severity of inflammation, which is consistent with previous studies showing that mice treated with an anti-CD226 polyclonal antibody (pAb) *in vivo* were resistant to EAE development and progression (27).

Next, we observed fewer mononuclear cell infiltrates in the spinal cords and brains of *Cd226*^−/−^ mice than in WT mice at the peak of EAE (Figure 2B). LFB staining results revealed that demyelination in the lumbar region of the spinal cord was lower in *Cd226*^−/−^ mice than that observed in WT mice (Figure 2C), and the haematoxylin-eosin (H&E) staining results showed a reduction in lymphocyte infiltration in the lumbar region of the spinal cord of *Cd226*^−/−^ mice during the peak of EAE (Figure 2D). EAE is a myelin-specific autoreactive Th17 and Th1 cell-mediated inflammatory disease (28), and Th17 cells have been reported to have a greater pathogenic impact on the development of EAE than Th1 cells (29). Immunohistochemical analysis showed that infiltration of IFN-γ^+^ cells in the brains of *Cd226*^−/−^ mice was comparable to that observed in WT mice at the peak of EAE (Supplemental Figure 2B). We then examined IL-17A expression by immunohistochemical staining and detected fewer IL-17A^+^ cells in the lumbar spinal cord (cross-sections) of *Cd226*^−/−^ EAE mice compared to that observed in WT EAE mice (Figure 2E). Because the longitudinal sections clearly highlight the increased extravasation from the spinal cord fluid compared with that observed in the transversal sections at the level of the ependymal channel, we also used the transversal sections of lumbar spinal cords to identify inflammatory infiltrates. Notably, we also observed decreased IL-17A^+^ cell infiltration in the lumbar spinal cords of *Cd226*^−/−^ mice during EAE (Supplemental Figure 3A), indicating that CD226-deficient mice during EAE had reduced immune activation compared with WT mice during EAE. From these results, we presumed that the CD226 deficiency in mice led to decreased EAE susceptibility, in part because of decreased inflammatory Th17 cell infiltration into the CNS. Because the results of our previous study showed that CD226 is robustly expressed in the brain (30), we next investigated whether deletion of CD226 in mice reduces the number of cells infiltrating the brain and damage. We observed that both infiltrating cells and demyelination were reduced in the brain tissue of *Cd226*^−/−^ compared with that observed in WT mice (Supplemental Figures 3B,C). RORγt is a transcription factor that is unique to Th17 cells and directs the polarization of naïve CD4^+^ T cells to Th17 cells (31). The transcription factor Foxp3 is a key factor in the regulation of Treg-mediated immunosuppressive responses (32). Immunofluorescence staining results showed that the amount of RORγt protein was reduced (Figure 2F) and that Foxp3 protein was increased in the brains of *Cd226*^−/−^ mice compared with that observed in their WT counterparts during the peak inflammation period of EAE (Figure 2G). These data collectively suggest that the deletion of CD226 may lead to a reduced inflammatory response in mice that is associated with high numbers of Tregs and reduced infiltration of Th17 cells in the CNS.

### Decreased Numbers of Th17 Cells and Increased Numbers of Tregs in the Spleens of *Cd226*^–/–^ EAE Mice

**Figure 3:**
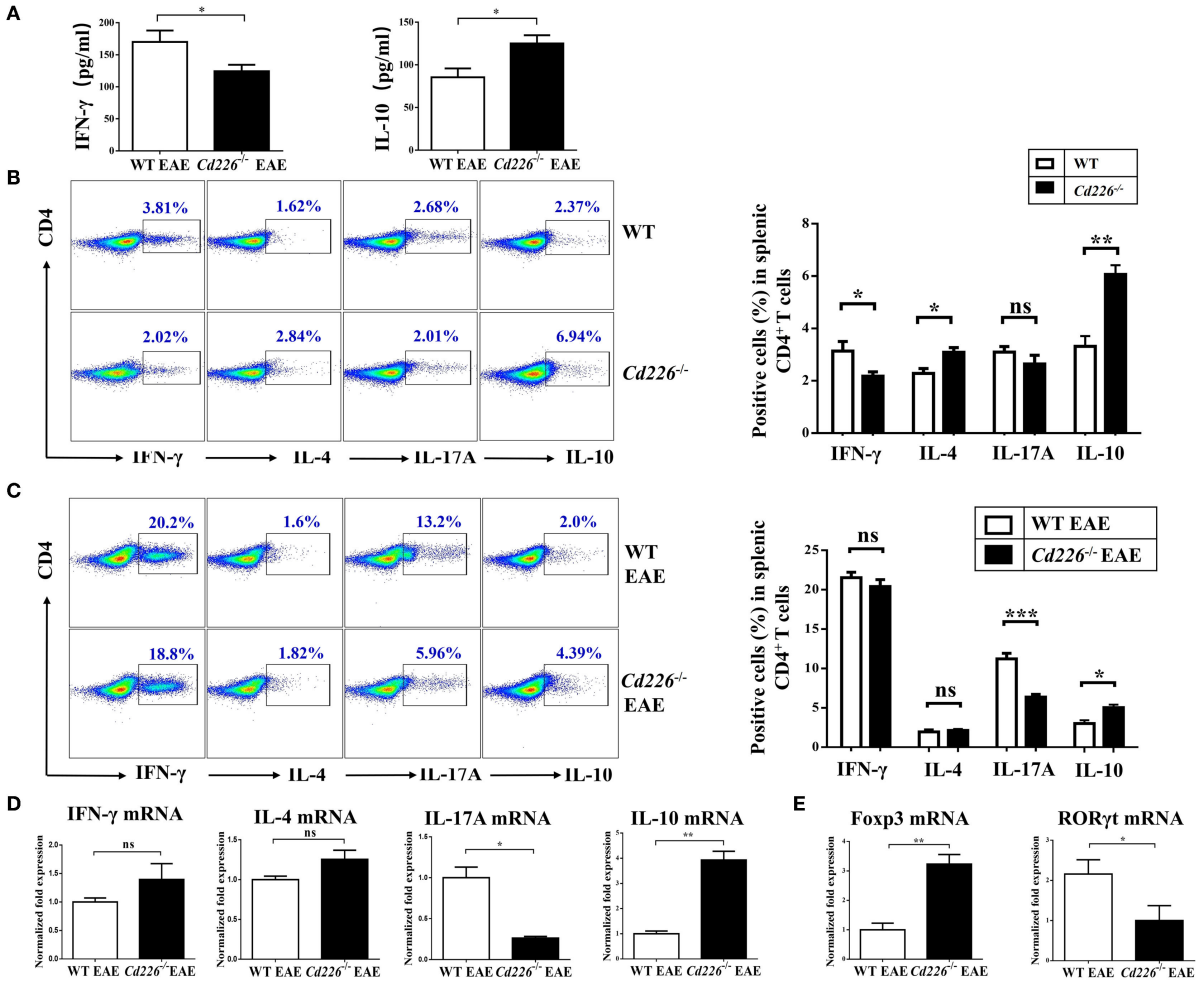
Lower levels of Th17 cells and higher levels of Tregs were observed in the spleen of *Cd226*^−/−^ EAE mice. **(A)** The plasma levels of IFN-γ and IL-10 in *Cd226*^−/−^ and WT mice during EAE pathogenesis were assessed by ELISA (*n* = 8). **(B)** Protein expression of IFN-γ, IL-4, IL-17A and IL-10 in sorted CD4^+^ T cells from splenocytes in *Cd226*^−/−^ mice and WT littermates was assessed by intracellular staining. The numbers in each quadrant show the percentage of the relevant cell population (*n* = 8). **(C)** Splenocytes were stimulated *in vitro* with PMA (phorbol myristate acetate), ionomycin and brefeldin A in 10% FBS/RPMI 1,640 for 6 h. FCM data analysis of CD4^+^ T cell subsets for cytokines released from the spleens of *Cd226*^−/−^ or WT mice was conducted on days 15–18 after immunization with MOG_35−55_ (gated on total CD4^+^ T cells, with frequencies on the left). The proportions of Th1, Th2, Th17 cells and Tregs were determined by intracellular cytokine staining (*n* = 6). **(D)** The relative mRNA levels of IFN-γ, IL-4, IL-17A, and IL-10 in CD4^+^ T cells isolated from the spleens of *Cd226*^−/−^ or WT mice at days 15–18 after immunization with MOG_35−55_ were determined by RT-qPCR (*n* = 6). **(E)** The relative mRNA levels of Foxp3 and RORγt in CD4^+^ T cells isolated from the splenocytes of WT or *Cd226*^−/−^ mice on days 15–18 after immunization with MOG_35−55_ were determined by RT-qPCR (*n* = 6). The results represent at least three independent experiments **(A–E)**. The data are presented as the mean ± SEM. ^ns^*P* > 0.05, *P < 0.05, **P < 0.005, ***P < 0.001.

IFN-γ is a pro-inflammatory cytokine that can enhance the degree of inflammation in EAE (33). To investigate whether CD226 deficiency could inhibit IFN-γ production, the plasma level of IFN-γ in *Cd226*^−/−^ mice was examined in the EAE model. The level of IFN-γ in *Cd226*^−/−^ mice was apparently lower than that observed in WT mice during EAE pathogenesis (Figure 3A). Moreover, mice deficient in CD226 showed increased levels of the inhibitory cytokine IL-10 in plasma (Figure 3A). To investigate whether CD226-deficient mice were prone toward a loss of homeostasis of CD4^+^ T cell subsets in the periphery, we compared representative cytokines produced by CD4^+^ T cell subsets between *Cd226*^−/−^ and WT mice under healthy conditions (Figure 3B). IFN-γ and IL-17A are the primary effector cytokines of Th1 and Th17 subsets, respectively. The results showed that the IFN-γ^+^ CD4^+^ T cell compartment was slightly decreased, while that of the IL-17A^+^ CD4^+^ T cell subset was normal in *Cd226*^−/−^ mice. In contrast, the levels of IL-10 and IL-4, the signature chemokines for Tregs and Th2 cells, respectively, were clearly increased in the CD4^+^ T cells of *Cd226*^−/−^ mice (Figure 3B). These results collectively indicated an intrinsic role of CD226 in regulating cytokine production in CD4^+^ T cell subsets.

The reduction of EAE severity in *Cd226*^−/−^ mice prompted us to examine whether CD226 is involved in regulating commitment to the CD4^+^ T cell lineage. Therefore, we investigated how these CD4^+^ T sub-populations were affected by CD226 deletion under EAE conditions. At days 15–18 after MOG_35−55_ immunization, the expression levels of IFN-γ, IL-4, IL-17, and IL-10 in CD4^+^ T cells sorted from the splenocytes of *Cd226*^−/−^ or WT mice were evaluated by flow cytometry (Figure 3C). During the peak stage of EAE progression, although the production of IFN-γ in CD4^+^ T cells was greatly enhanced in both *Cd226*^−/−^ and WT mice compared with that observed in healthy mice, we did not observe a significant difference in the level of this cytokine between *Cd226*^−/−^ and WT mice. We further evaluated whether CD226 deficiency could affect cytokine production by Th2 cells and observed that CD4^+^ T cells from *Cd226*^−/−^ mice had an IL-4 response to MOG_35−55_ that was comparable to that of the WT littermate controls. Th17 cells have been reported to be major factors involved in the development and progression of EAE (34). Notably, we observed that number of CD4^+^ IL-17A^+^ T cells was not increased in *Cd226*^−/−^ mice but was sharply increased in WT mice. The increased production of IFN-γ and IL-17A in the WT mice during EAE largely accounted for the exacerbated Th1 cells and Th17 autoimmune response cells, highlighting the crucial role of CD226 in CD4^+^ T cell participation in Th1 and Th17 reactions. In contrast to the role of Th17 cell-related IL-17A in exacerbating EAE pathogenesis, IL-10 is a defining anti-inflammatory cytokine for Tregs, playing an essential role in negatively regulating autoimmune responses (33). Our results showed an increase in IL-10 production from CD4^+^ T cells of *Cd226*^−/−^ mice compared with that observed in WT mice during EAE. Thus, we speculated that the Tregs of *Cd226*^−/−^ mice are activated under EAE conditions (Figure 3C). Taken together, the alleviation of EAE by the deletion of CD226 in mice likely occurred by restoring the balance of Tregs/Th17 cells. To further verify these results at the mRNA level, we sorted CD4^+^ T cells from the spleens of *Cd226*^−/−^ and WT mice at the peak of EAE and detected the mRNA expression levels of the cytokines IFN-γ, IL-4, IL-17A, and IL-10 and the transcription factors RORγt and Foxp3 by RT-qPCR. Consistent with the flow cytometry (FCM) results, we observed a decrease in IL-17 but a marked increase in IL-10 levels, with no significant changes observed in IFN-γ and IL-4 in *Cd226*^−/−^ EAE mice (Figure 3D). We also observed that the level of Foxp3 mRNA was clearly increased and that of RORγt mRNA was decreased in splenic CD4^+^ T cells from *Cd226*^−/−^ EAE mice (Figure 3E). From these results, we concluded that deletion of CD226 decreased the level of Th17 cells but facilitated Tregs in the spleens of *Cd226*^−/−^ mice during EAE.

### *Cd226*^–/–^ Tregs Are Highly Proliferative and Suppressive *in vivo*

**Figure 4:**
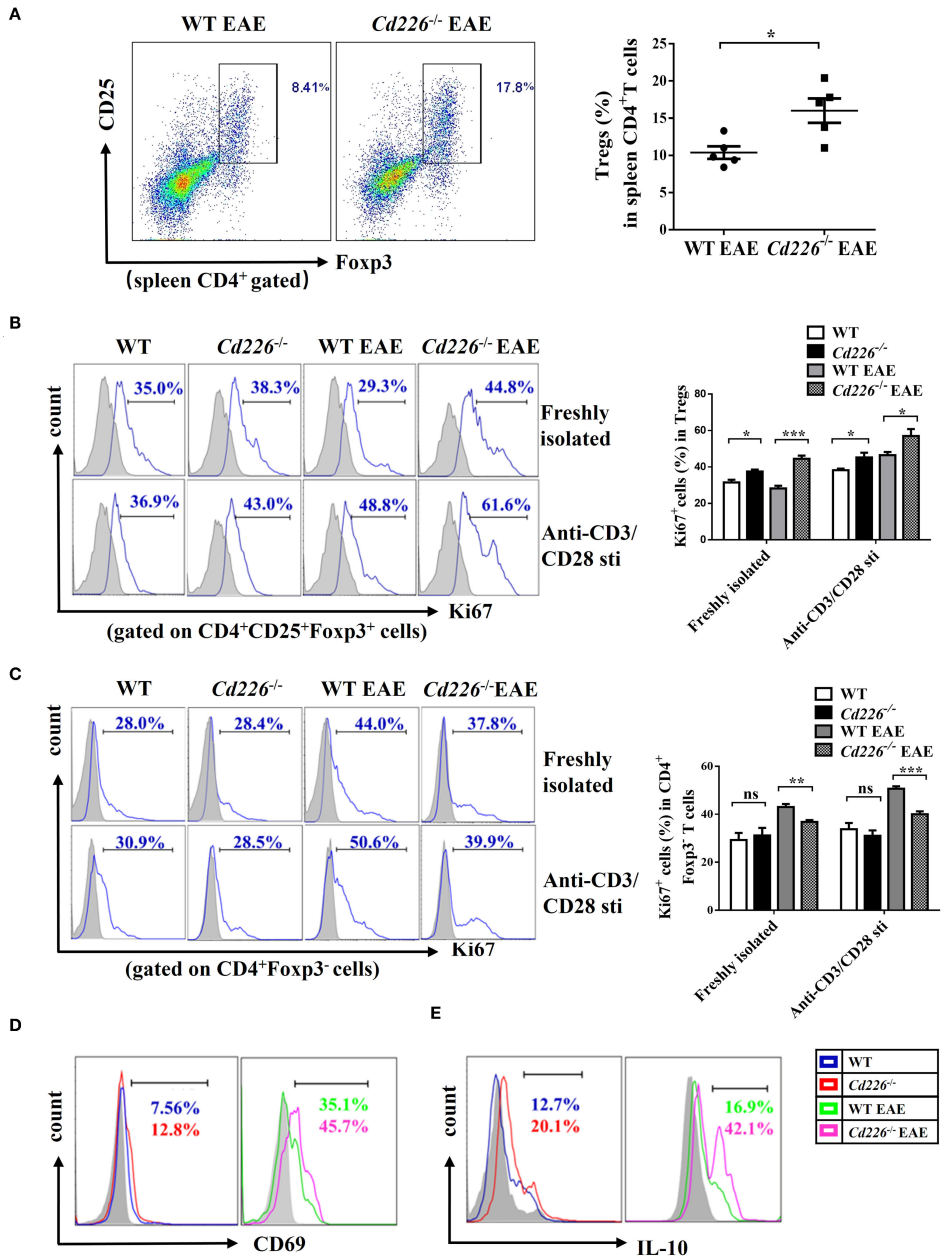
*Cd226*^−/−^ Tregs are highly proliferative and suppressive *in vivo*. **(A)** The percentages of Tregs in spleen CD4^+^ T cells from *CD226*^−/−^ and WT littermate mice on days 15–18 after treatment with MOG_35−55_ (*n* = 6). **(B)** Percentage of Ki67^+^ cells in CD4^+^Foxp3^+^ Tregs freshly isolated or after 3 h of culturing with anti-CD3 (5 μg/ml)/anti-CD28 (5 μg/ml) from spleen cells of *Cd226*^−/−^ and WT mice on days 15–18 after MOG_35−55_ immunization by FCM (*n* = 3). **(C)** Percentage of Ki67^+^ cells in CD4^+^Foxp3^−^ Tconv cells freshly isolated or after 3 h of culturing with anti-CD3 (5 μg/ml)/anti-CD28 (5 μg/ml) from splenocytes of *Cd226*^−/−^ and WT mice at the peak of EAE were detected by FCM (*n* = 3). Expression of CD69 **(D)** or IL-10 **(E)** in splenic CD4^+^ CD25^+^ Foxp3^+^ Tregs from *Cd226*^−/−^ and WT mice under healthy conditions or EAE pathogenesis were detected by FCM (*n* = 6). The data are presented as the mean ± SEM. ^ns^*P*> 0.05, *P < 0.05, **P < 0.005, ***P < 0.001.

At the peak of EAE, we observed that the percentage of Tregs in splenic CD4^+^ T cells was increased upon CD226 deletion (Figure 4A). To elucidate the mechanism responsible for the higher percentage of Tregs in CD4^+^ T cells from *Cd226*^−/−^ EAE mice, we further examined Treg proliferation and suppression *in vivo*. Freshly isolated Tregs from *Cd226*^−/−^ healthy mice expressed slightly higher percentages of the proliferation marker Ki67 than WT mice. At 15–18 days after immunization with MOG_35−55_, the expression of Ki67 in the freshly isolated Tregs from WT mice was clearly decreased, while that of the Tregs from *Cd226*^−/−^ mice was not. Notably, under EAE conditions, the expression of Ki67 in *Cd226*^−/−^ Tregs was higher than that observed in WT Tregs, indicating an elevated level of *Cd226*^−/−^ Treg proliferation (Figure 4B). We next wanted to determine whether the deletion of CD226 affected the proliferation of Tregs after TCR stimulation. To this end, we assessed Ki67 expression in Tregs with anti-CD3/anti-CD28 antibodies after 3 h of culturing. Compared with their WT EAE counterparts, the Tregs from *Cd226*^−/−^ EAE mice showed higher expression of Ki67 (Figure 4B). In contrast, freshly isolated Tconv cells from *Cd226*^−/−^ EAE mice expressed lower levels of Ki67 than their WT counterparts during EAE pathogenesis. This pattern was particularly notable after anti-CD3/anti-CD28 stimulation for 3 h. These results demonstrated that CD226 depletion led to high proliferation of Tregs under EAE conditions, whereas Tconv cells were less activated than the WT mice (Figure 4C).

To further confirm these results, we examined the activation status of Tregs from *Cd226*^−/−^ mice during EAE. The level of CD4^+^Foxp3^+^ Tregs from *Cd226*^−/−^ mice with an acute activated CD69 phenotype was increased compared with that observed in WT mice, especially during EAE pathogenesis (Figure 4D). Based on these results, we explored whether this enhanced proliferation in Tregs was associated with dysregulated cytokine production. IL-10 has been demonstrated to play an important role in the suppression of Th17 cell-mediated inflammation (35). Therefore, we analyzed the production of IL-10 from Tregs in *Cd226*^−/−^ mice under healthy conditions and during EAE inflammatory conditions. The results showed that Tregs in *Cd226*^−/−^ mice generated large amounts of IL-10, and this expression was notably increased during EAE inflammation (Figure 4E). TGF-β plays a pivotal role in regulation of the differentiation and function of Tregs (36). So we next magnetically sorted Tregs (cell purity ≥94%) (Supplemental Figure 4A) from the spleens of *Cd226*^−/−^ and WT mice at the peak of EAE and detected the mRNA expression level of TGF-β. The results showed that TGF-β mRNA was largely normal in *Cd226*^−/−^ EAE mice compared with WT EAE mice, suggesting that the TGF-β may not involve in the enhanced function of Tregs (Supplemental Figure 4B). Taken together, these results indicated that the deletion of CD226 led to enhanced Treg proliferation and increased IL-10 production.

### *Cd226^−/−^* iTregs Are Highly Proliferative and Suppressive *in vitro*

**Figure 5:**
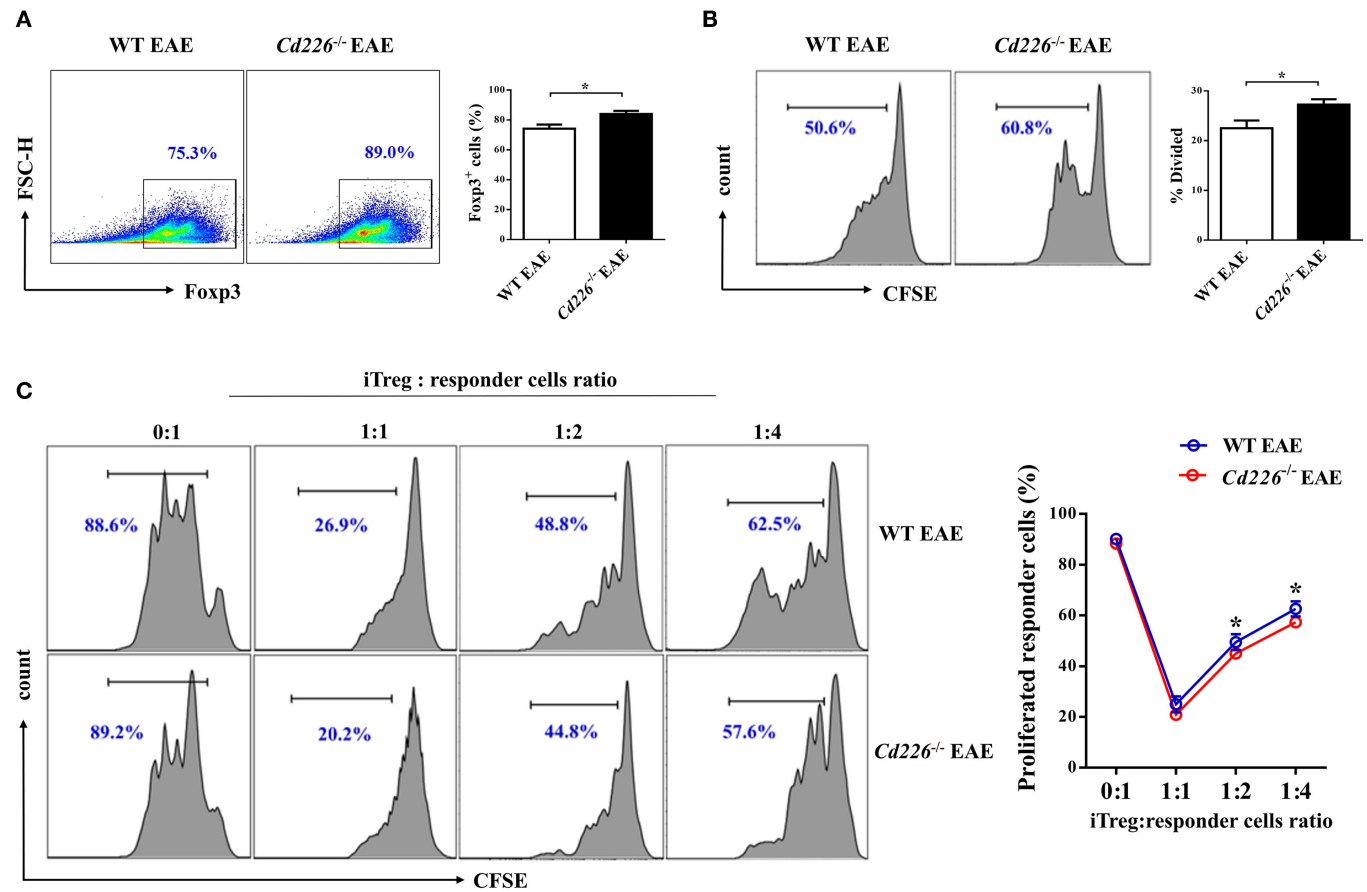
*Cd226*^−/−^ iTregs are highly proliferative and suppressive *in vitro*. **(A)** Naïve CD4^+^ T cells from WT and *Cd226*^−/−^ mouse spleens from days 15–18 after MOG_35−55_ immunization were magnetically sorted. Then, the cells were cultured under iTreg polarizing conditions [plate-coated anti-CD3 (3 μg/ml), soluble anti-CD28 (5 μg/ml), IL-2 (2 ng/ml)] and recombinant TGF-β (5 ng/ml), and Foxp3 expression was measured by FCM after 3 days of culture (*n* = 6). **(B)** The proliferation of iTregs was assessed by FCM. iTregs were labeled with CFSE and cultured in the presence of anti-CD3 (5 μg/ml) and anti-CD28 (5 μg/ml) for 3 days, and the proliferative capacity was detected 3 days later (*n* = 6). **(C)** The suppressive capacity of iTregs was detected by FCM. CD4^+^CD25^+^ iTregs induced from naïve CD4^+^ T cells of WT or *Cd226*^−/−^ mice were sorted and co-cultured with the indicated ratios of CFSE-labeled responder CD4^+^ T cells from WT mice for 5 days in the presence of anti-CD3 (5 μg/ml) and anti-CD28 (5 μg/ml) (*n* = 3). The results represent three independent experiments **(A–C)**. The data are presented as the mean ± SEM. ^ns^*P* > 0.05, *P < 0.05.

Although the deletion of CD226 facilitated Treg proliferation and IL-10 production during inflammatory conditions *in vivo*, the relationship between CD226 deletion and Treg *in vitro* function remains unclear. To investigate whether the deletion of CD226 could also influence the suppressive function of Tregs *in vitro* during EAE, we decided to preliminarily investigate the abilities of iTreg cells. We isolated naïve CD4^+^ T cells (CD4^+^CD62L^+^) from WT EAE and *Cd226*^−/−^ EAE mice and cultured them for 3 days under Treg-polarizing conditions. The iTreg proliferation was assessed by FCM after incubating overnight with plate-bound anti-CD3 at 4°C and soluble anti-CD28 stimulation for 3 days *in vitro*. Our results showed that CD226-deficient naïve CD4^+^ T cells had an enhanced ability to induce iTregs comparable to that of WT mice (Figure 5A). Moreover, the rate of CFSE-labeled iTreg proliferation was higher than that observed in WT mice (Figure 5B), indicating that the iTregs induced from *Cd226*^−/−^ EAE mice remained energetic *in vitro*, to an even higher extent than those from WT EAE mice. To determine whether the loss of CD226 in iTregs is linked to apoptosis, we stained iTregs with PI and annexin V and observed a comparable percentage of apoptotic cells between WT and *Cd226*^−/−^ iTregs, indicating that CD226-deficient iTregs did not display decreased apoptosis relative to their CD226-sufficient counterparts (Supplemental Figure 4C). Next, to directly evaluate whether the immunosuppressive activity of CD226-deficient iTreg cells was affected *in vitro*, we used an *in vitro* suppression assay. Responder CD4^+^ T cells were magnetically sorted from healthy WT mice and labeled with CFSE. The iTregs induced from naïve CD4^+^ T cells of *Cd226*^−/−^ EAE or WT EAE mice were then cultured with varying amounts (as indicated) of labeled responder CD4^+^ T cells in the presence of anti-CD3/CD28 for 5 days, after which the responder cell proliferation was determined. The results confirmed that CD226-deficient iTregs highly suppressed responder cell function *in vitro* and showed an enhanced ability to suppress responder cell proliferation under TCR-stimulated conditions compared with the iTregs induced in WT EAE mice (Figure 5C). These results further demonstrated that the deletion of CD226 facilitated the suppressive capacity of Tregs.

### Akt and Erk Signaling Pathways Are Involved in *Cd226*^–/–^ Treg Proliferation and Function

**Figure 6:**
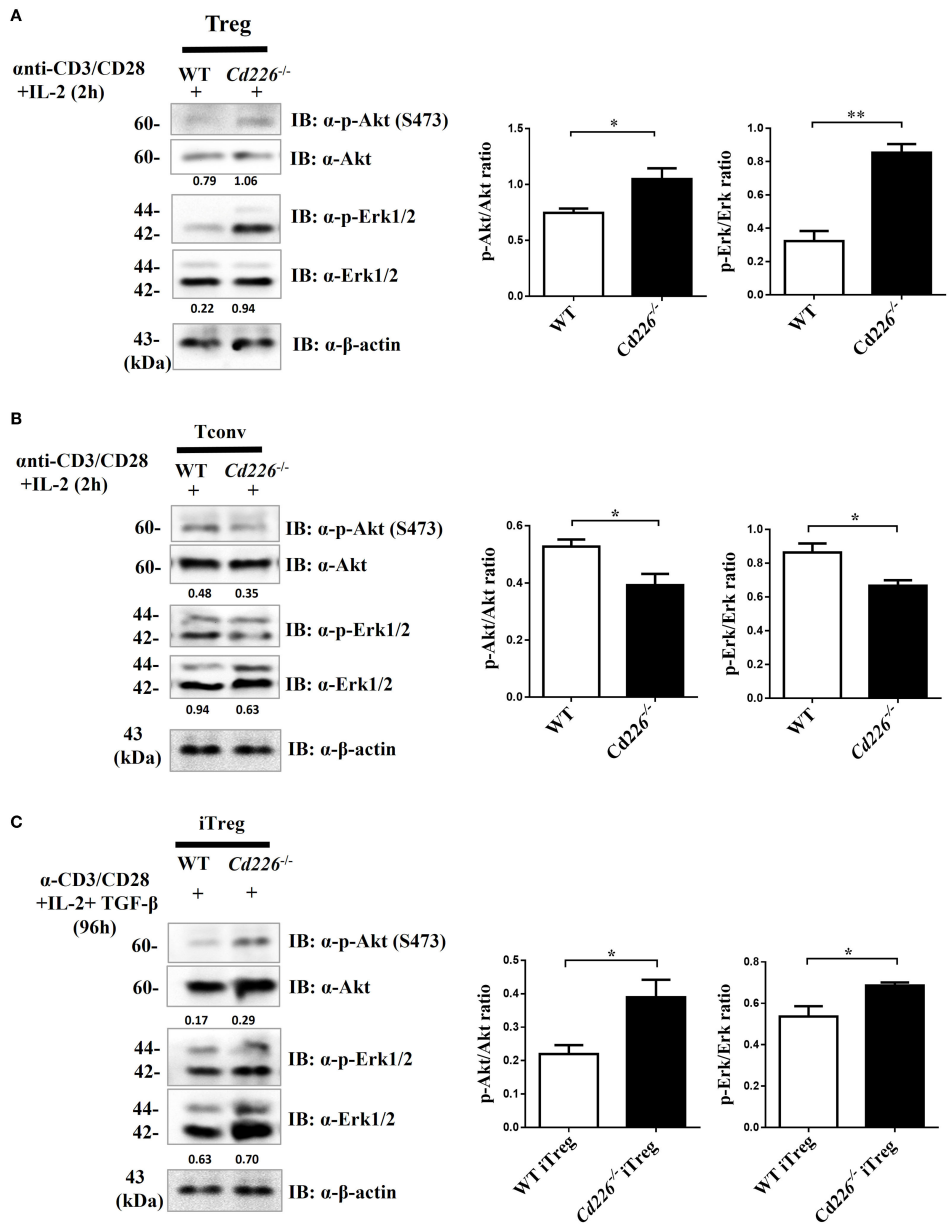
Higher levels of Akt and Erk signaling occur in *Cd226*^−/−^ Tregs. Splenic Tregs **(A)** and Tconv cells **(B)** magnetically isolated from WT or *Cd226*^−/−^ mice were stimulated with anti-CD3 (5 μg/ml), anti-CD28 (5 μg/ml) and IL-2 (2 ng/ml) for 2 h, after which p44/42 (Erk1/2), phospho-p44/42 MAPK (Erk1/2), Akt and phosphorylated-Akt (Ser473) were detected by Western blotting (*n* = 6). **(C)** Naïve CD4^+^ T cells magnetically sorted from WT and *Cd226*^−/−^ mouse spleen were cultured under iTreg cell polarizing conditions for 96 h [plate-bound anti-CD3 (3 μg/ml) plus soluble anti-CD28 (5 μg/ml), IL-2 (2 ng/ml) and recombinant TGF-β (5 ng/ml)]. The levels of Erk1/2, phosphorylated-Erk (p-Erk1/2), Akt and phosphorylated-Akt (p-Akt) (Ser473) in iTregs was detected by Western blotting (*n* = 6). The Western blot results were quantified by ImageJ analysis and are shown as the p-Erk/Erk or p-Akt/Akt ratios. The results represent three independent experiments **(A–C)**. The data are presented as the mean ± SEM. *P < 0.05, **P < 0.005.

Previous studies have shown that Akt and Erk signaling pathways are involved in regulating T cell survival, proliferation, and differentiation (37, 38). To address whether the loss of CD226 can affect the Akt and Erk signaling pathways, which are associated with Treg proliferation and suppression, we observed the biochemical mechanisms of CD226 *in vivo* in sorted Tregs and *in vitro*-generated iTregs by assessing Akt and Erk signaling. After stimulating freshly isolated Tregs with anti-CD3/CD28 in the presence of IL-2 for 2 h, we observed that *Cd226*^−/−^ Tregs showed higher levels of phosphorylated-Akt (ser473) and phosphorylated p44/42 (Erk1/2) than WT Tregs (Figure 6A). In contrast, the *Cd226*^−/−^ Tconv cells showed decreased activation of Akt signaling and reduced activation of Erk signaling compared with that observed for WT Tconv cells (Figure 6B). Next, we evaluated whether the enhanced proliferation of *Cd226*^−/−^ iTregs *in vitro* was associated with activation of the Akt and Erk signaling pathways by assessing the phosphorylation levels of Akt (ser473) and p44/42 (Erk1/2) in iTregs differentiated from naïve WT and *Cd226*^−/−^ CD4^+^ T cells for 96 h. As expected, the *Cd226*^−/−^ iTregs exhibited higher levels of phosphorylation of both Akt and Erk than WT iTregs (Figure 6C). These results confirmed that the enhanced proliferation of *Cd226*^−/−^ Tregs may be associated with activated Akt and Erk signaling.

## Discussion

Several lines of evidence have demonstrated that CD226 plays a crucial role in the formation of immune synapses, and it is primarily considered to be an active receptor of NK and T cells (3, 39). The results of our previous studies demonstrated that mice treated with an anti-CD226 pAb *in vivo* have markedly decreased EAE susceptibility (27), in agreement with recent data showing that CD226 is involved in the pathogenesis of autoimmune diseases (8, 9). EAE is caused by the breakdown of self-tolerance and eventually leads to myelin-reactive CD4^+^ T cell infiltration of the CNS to mediate neuronal inflammation (11, 29). Thus, we focused on the function of CD226 in CD4^+^ T subset cells during EAE. Prior studies showed that the murine CD226/Ig fusion protein can inhibit the production of thymocytes and speculated that CD226 may be involved in the early stage of T cell development (40). Studies in rodents have shown that knockdown of CD226 in naïve CD4^+^ T cells results in decreased T-bet expression and increased GATA3 expression, as well as demonstrating that CD226 blockade with a neutralizing Ab *in vitro* can efficiently downregulate IL-17 secretion (41). Because CD4^+^ T cells play a central role in autoimmunity, and CD226 promotes self-reactive CD4^+^ T cell activation in the autoimmune response (8, 42), we considered whether CD226 functions by altering the balance of CD4^+^ T cell subsets involved in the pathogenesis of EAE. Therefore, we developed an EAE mouse model to better understand the unique functions and mechanisms of CD226 in CD4^+^ T cell lineages under pathological conditions. Tregs are known to suppress the response of myelin-reactive CD4^+^ T_eff_ and are pivotal in regulating CNS inflammation during EAE (43). Several studies have reported disruptions in the numbers or function of Tregs involved in the pathogenesis of EAE (16), but the factors affecting the stability and function of Tregs are poorly understood. In our study, we observed an increased level of CD226 expression in spleen CD4^+^ T cells and CD4^+^ Foxp3^−^ Tconv cells; however, CD226 expression was not upregulated in Tregs under EAE conditions. These observations suggest a potentially distinct role for CD226 in controlling CD4^+^ T cell lineages, especially in Tregs. Despite evidence of the genetic associations of CD226 with autoimmune diseases and dysfunctional Tregs (7, 44, 45), as well as studies noting that CD226^+^TIGIT^−^ Tregs are associated with reduced suppressive capacity (46), the exact molecular mechanism and function role of CD226 in Tregs is poorly understood.

To examine whether CD226 mediates the auto-reactive CD4^+^ T cell response and the immunosuppressive function of Tregs during EAE, we compared the disease development, demyelinating conditions, inflammatory process and distribution of CD4^+^ T subsets in CNS tissue in *Cd226*^−/−^ EAE and WT EAE mice. We observed that CD226 deletion delayed the onset and alleviated the development of EAE. *Cd226*^−/−^ mice tended to show reduced body weight loss at days 4–6 and 20–28 after MOG_35−55_ immunization, but unfortunately, we did not observe significant differences between the two groups during EAE. We speculate that this result may have been due to the short peak period of EAE and the gradual dampening of disease development after the acute phase. Additionally, the infiltration of inflammatory cells into the CNS was decreased. We also observed a decreased number of RORγt^+^ cells and an increasing number of Foxp3^+^ cells that infiltrated into the CNS tissue in *Cd226*^−/−^ EAE mice. These results collectively suggest that the deletion of CD226 may lead to a reduced inflammatory response in mice, which is most likely associated with maintaining the Treg/Th17 immune balance in the CNS. Recently, the imbalance between Th17 cells and Tregs has been considered to be an important paradigm in the development and pathogenesis of EAE (47). Several studies have demonstrated that the migration of Th17 cells into the CNS is the key pathogenic process in EAE mice (34, 48) and that Foxp3 deficiency is closely associated with spontaneous autoimmunity development (13, 49, 50). CD226 is a co-stimulatory molecule that plays an important role in the activation, expansion and function of Th1 cells (51). Moreover, CD226/CD155 interaction has been demonstrated to regulate the pro-inflammatory (Th1 and Th17) and anti-inflammatory (Th2) balance (41). Based on these findings and our results, we speculated that CD226 is a crucial molecule that regulates auto-reactive Th17 cells and Treg immune responses in EAE. However, additional studies are needed to elucidate the effects of CD226 blockade Ab treatment on the CNS tissue inflammatory responses during EAE pathogenesis.

Based on their distinct cytokine profiles, CD4^+^ T subsets have different functional properties (52). IL-17 secreted by Th17 cells has been shown to have a pathogenic role in promoting and enhancing autoimmune tissue injuries (53). IL-10, an important mediator with anti-inflammatory properties, is primarily produced by Tregs and can inhibit Th17 cell differentiation in the CD4^+^ T cell population. We next assessed the frequency and numbers of the CD4^+^ T subsets of spleen cells at the peak of EAE to understand whether similar changes occur in the peripheral immune organs. In our study, we observed that deletion of CD226 increased the expression of IL-10 and decreased that of IL-17A, whereas it did not notably alter IFN-γ and IL-4 expression in CD4^+^ T cells during EAE. The administration of IL-10 has been demonstrated to suppress EAE progression (54), and the induction of EAE in IL-17^−/−^ mice was shown to reduce disease severity and promote early recovery (55). Based on our results, we speculate that deletion of CD226 does not greatly affect the Th1 and Th2 cellular immune response but rather alters the balance of Treg/Th17 cells in the EAE model. These results are consistent with a report showing that CD226 costimulatory signaling is associated with LFA-1 to amplify TCR signaling through VAV1, further promoting IL-17 production by CD4^+^ T cells (56). Additionally, our observation further confirmed that CD226 may play an important role in the activation and effector functions of Th17 cells under EAE inflammatory conditions.

The stable expression of Foxp3 and potent suppressive capacity are two basic characteristics that are maintained in the Treg lineage (57). Another major finding of our study is that deletion of CD226 led to the upregulation of Treg proliferation and enhanced the suppressive function of Tregs during EAE. Recently, studies have demonstrated that CD226 is involved in the function and stability of Tregs (20) and that CD226^+^TIGIT^−^ Tregs exhibit enhanced methylation in the Treg-specific demethylated region (TSDR) (46), indicating that CD226 may regulate Treg function. In our study, the Tregs in *Cd226*^−/−^EAE mice expressed high levels of the proliferation marker Ki67 and the acute activation marker CD69. Tregs are typically stable and able to retain lineage characteristics *in vivo*. However, during inflammation or other pathological conditions, Tregs may lose Foxp3 expression and then exhibit a reduced suppressive capacity (58). Our results showed that the CD226 deficiency maintained iTreg functions during EAE pathogenesis. We observed that the proliferation of responder CD4^+^ T cells was decreased after co-culturing with iTregs, indicating that CD226 deletion promoted the suppressor capacity of iTreg cells *in vitro*. Many studies have demonstrated that decreased IL-10 production by Tregs is linked to MS (16). Treg-secreted IL-10 plays an essential role in the negative regulation of autoimmune responses. Notably, we observed that a significant increase in the expression of IL-10 in CD226-deficient Tregs at the peak of EAE, indicating that the deletion of CD226 maintained the suppressive capacity of Tregs, partially through the promotion of IL-10 signaling.

The Akt and Erk signaling pathways function downstream of TCR and CD28 signaling and are important in the regulation of survival, growth, proliferation and differentiation processes in T cells under varying levels of TCR signaling stimulation (59, 60). Studies have reported a key role for the Akt pathway in the TCR-independent phase of thymic Treg generation (61) and BMP-2, 4, members of the TGF-β superfamily, promote Treg development through the Erk pathway (62). Moreover, Previous studies demonstrated that CD226 triggers signals involving activation of Akt and Erk (63), and the engagement of human CD226 by anti-CD226 antibodies failed to trigger activation of Akt and Erk (64). To determine whether the increased proliferation of *Cd226*^−/−^ and *Cd226*^−/−^ iTregs was associated with these pathways, we measured the expression of the Akt and Erk kinases *in vitro*. Our results demonstrated that *Cd226*^−/−^ and *Cd226*^−/−^ iTregs stimulated with anti-CD3/CD28 plus IL-2 exhibited Akt and Erk pathway activation, but *Cd226*^−/−^ Tconv cells did not. The mechanistic target of rapamycin (mTOR) is a nutrient sensing kinase that functions downstream of Akt (65). mTOR is now well recognized as an important regulator of CD4^+^ T subsets (66) that influences the development of autoimmune diseases such as MS and EAE (67, 68). mTOR has two distinct signaling complexes, mTOR1 and mTOR2, which have pivotal roles in regulating the differentiation of CD4^+^ T subsets (69). Our results revealed a high proportion of inflammatory cell infiltration into the CNS in WT but not *Cd226*^−/−^ mice at the peak of EAE, and *Cd226*^−/−^ Tconv cells showed decreased activation of Akt signaling. Based on these results, we speculated that the deletion of CD226 in mice blocks the Akt pathway in Tconv cells, which especially influences mTOR1 activation. Rapamycin specifically blocks mTOR1 activation and effectively inhibits T cell proliferation (70), and evidence from mouse studies and clinical trials has demonstrated that blockade of mTOR1 with rapamycin ameliorates the development of EAE and exerts beneficial effects in relapsing/remitting MS (67, 68). These observations correlate with our findings indicating that the deletion of CD226 blocks mTOR1 activation to prevent the differentiation of Th1 and Th17 cells. Keever Taylor and colleagues reported that rapamycin-treated Treg cultures from MS patients had enhanced suppressor functions and contained high levels of CD4^+^Foxp3^+^ cells compared with healthy controls, suggesting that blockade of mTOR1 promotes Treg development and function (71). In our studies, we observed the *Cd226*^−/−^ Tregs had increased Akt activation. Considering the distinct STAT activation functions of mTOR1 and mTOR2 (69, 72), it is possible that mTOR2 or another pathway may be involved in boosting the proliferation and function of *Cd226*^−/−^ Tregs. Thus, our results indicate that CD226 deletion facilitates the proliferation of Tregs and downregulates the activation of self-reactive CD4^+^ T cells, partially through activation of the Akt and Erk kinases. These findings are partly consistent with a report showing that blockade of the Erk pathway markedly attenuates Foxp3 expression in TGF-β-primed CD4^+^ T cells (37).

Taken together, the results of our study have revealed that CD226 acts as an essential molecule that influences the CD4^+^ T subset-mediated immune response, particularly the autoimmune reactions of differentially regulated Tconv and Tregs. Deletion of CD226 could enforce Treg differentiation and proliferation as well as Treg-mediated repression of Th17 responses. Multiple mechanisms are involved in the maintenance of the suppressive functions of Tregs in *Cd226*^−/−^ mice during EAE, such as the sustained expression of Foxp3, the production of high levels of the anti-inflammatory cytokine IL-10 and the activation of the Akt and Erk kinases. Nevertheless, an understanding of the CD226-mediated regulation of Treg immune functions is far from complete. Our future studies will utilize a CD226 blocking antibody and conditional CD226 knockout mice to explore the crosstalk between CD226 and intracellular molecule signaling in Tregs. This information will provide support for the therapeutic applications of CD226 blockade in inflammatory diseases.

## Data Availability Statement

All datasets generated for this study are included in the article/Supplementary Material.

## Ethics Statement

The animal study was reviewed and approved by the Scientific Investigation Board of the Fourth Military Medical University, Xi'an (permit number XJYYLL-2014433).

## Author Contributions

LC and BJ designed experiments. NW, SL, JJ, and JX carried out experiments. ZL and HZ analyzed experimental results. LF and HY analyzed FCM data. QM and FJ assisted with NW wrote the manuscript. JL and YS completed the immunohistochemical staining of the revised version. All authors contributed to the article and approved the submitted version.

## Conflict of Interest

The authors declare that the research was conducted in the absence of any commercial or financial relationships that could be construed as a potential conflict of interest.

## Supplementary Material

Supplemental Figure 1 Efficient CD226 deletion in *Cd226*^−/−^ mice. Total CD4^+^ T cells, Tregs (CD4^+^ CD25^+^ Foxp3^+^) and Tconv cells (CD4^+^ Foxp3^−^) were gated from the splenocytes of *Cd226*^−/−^ mice, and the expression levels of CD226 were detected by FCM (*n* = 8). The data are representative of four independent experiments. The numbers in each quadrant show the percentage of the relevant cell population.

Supplemental Figure 2 Characterization of WT EAE and *Cd226*^−/−^ EAE mice. **(A)** The body weight of *Cd226*^−/−^ and WT mice was monitored daily after immunization with MOG_35−55_ (*n* = 16). The results represent eight independent experiments. **(B)** IFN-γ expression in the brain on days 15–18 post-immunization was detected by immunohistochemical staining (*n* = 6), and the integrated optical density of IFN-γ was measured with ImageJ. The results represent three independent experiments. ^ns^*P* > 0.05.

Supplemental Figure 3 Lower levels of infiltrated cells and demyelination in the CNS of *Cd226*^−/−^ Mice. **(A)** IL-17A expression in the lumbar region of the spinal cord (longitudinal sections) at days 15–18 post-immunization was detected by immunohistochemical staining (*n* = 6) (scale bars indicate 100 and 50 μm for lower and higher magnification, respectively). **(B)** Histological analysis of lymphocyte infiltration in the brain tissue (cross-sections) of the indicated mice by H&E staining (scale bars indicate 50 μm for lower magnification, and 50 μm for higher magnification) (*n* = 6). **(C)** LFB staining of the brains (cross-sections) of WT and *Cd226*^−/−^ mice at the peak of EAE (*n* = 6). Demyelination of representative brain sections is indicated by loss of blue staining (scale bars indicate 1 mm and 100 μm for lower and higher magnification, respectively), and the total demyelinated area was measured with ImageJ. The results represent at least two independent experiments **(A–C)**. ^*^*P* < 0.05, ^**^*P* < 0.005.

Supplemental Figure 4 *Cd226*^−/−^ iTregs do not display decreased apoptosis. **(A)** The purity of Tregs before and after magnetic separation was determined by FCM (*n* = 6). **(B)** The relative mRNA expression levels of TGF-β in splenic Tregs from WT or *Cd226*^−/−^ mice during EAE were assessed by qRT-PCR. **(C)** Annexin V and PI staining of iTregs after polarization from WT and *Cd226*^−/−^ naïve CD4^+^ T cells for 3 days (*n* = 6). The results represent two or three independent experiments **(A–C)**. The numbers in each quadrant show the percentage of the relevant cell population.

Supplemental Table 1 The primers used for qRT-PCR in this study are listed.

## References

[1] Zeng H Zhang R Jin B Chen L. Type 1 regulatory T cells: a new mechanism of peripheral immune tolerance. Cell Mol Immunol. (2015) 12:566–71. 10.1038/cmi.2015.44 26051475 PMC4579656

[2] Jin B Scott JL Vadas MA Burns GF. TGF beta down-regulates TLiSA1 expression and inhibits the differentiation of precursor lymphocytes into CTL and LAK cells. Immunology. (1989) 66:570–6. 2541074 PMC1385159

[3] Shibuya K Shirakawa J Kameyama T Honda S Tahara-Hanaoka S Miyamoto A. CD226 (DNAM-1) is involved in lymphocyte function-associated antigen 1 costimulatory signal for naive T cell differentiation and proliferation. J Exp Med. (2003) 198:1829–39. 10.1084/jem.20030958 14676297 PMC2194159

[4] Sun C Molineros JE Looger LL Zhou XJ Kim K Okada Y. High-density genotyping of immune-related loci identifies new SLE risk variants in individuals with Asian ancestry. Nat Genet. (2016) 48:323–30. 10.1038/ng.3496 26808113 PMC4767573

[5] Deshmukh HA Maiti AK Kim-Howard XR Rojas-Villarraga A Guthridge JM Anaya JM. Evaluation of 19 autoimmune disease-associated loci with rheumatoid arthritis in a colombian population: evidence for replication and gene-gene interaction. J Rheumatol. (2011) 38:1866–70. 10.3899/jrheum.110199 21765104 PMC3170719

[6] Reinards TH Albers HM Brinkman DM Kamphuis SS van Rossum MA Girschick HJ. CD226 (DNAM-1) is associated with susceptibility to juvenile idiopathic arthritis. Ann Rheum Dis. (2015) 74:2193–8. 10.1136/annrheumdis-2013-205138 25057181

[7] Qiu ZX Zhang K Qiu XS Zhou M Li WM. CD226 Gly307Ser association with multiple autoimmune diseases: a meta-analysis. Hum Immunol. (2013) 74:249–55. 10.1016/j.humimm.2012.10.009 23073294

[8] Gross CC Schulte-Mecklenbeck A Runzi A Kuhlmann T Posevitz-Fejfar A Schwab N. Impaired NK-mediated regulation of T-cell activity in multiple sclerosis is reconstituted by IL-2 receptor modulation. Proc Natl Acad Sci USA. (2016) 113:E2973–82. 10.1073/pnas.1524924113 27162345 PMC4889377

[9] Ayano M Tsukamoto H Kohno K Ueda N Tanaka A Mitoma H. Increased CD226 expression on CD8+ T cells is associated with upregulated cytokine production and endothelial cell injury in patients with systemic sclerosis. J Immunol. (2015) 195:892–900. 10.4049/jimmunol.1403046 26109642

[10] Bar-Or A Hintzen RQ Dale RC Rostasy K Bruck W Chitnis T. Immunopathophysiology of pediatric CNS inflammatory demyelinating diseases. Neurology. (2016) 87:S12–19. 10.1212/WNL.0000000000002821 27572856

[11] King IL Dickendesher TL Segal BM. Circulating Ly-6C+ myeloid precursors migrate to the CNS and play a pathogenic role during autoimmune demyelinating disease. Blood. (2009) 113:3190–7. 10.1182/blood-2008-07-168575 19196868 PMC2665891

[12] Bettelli E Pagany M Weiner HL Linington C Sobel RA Kuchroo VK. Myelin oligodendrocyte glycoprotein-specific T cell receptor transgenic mice develop spontaneous autoimmune optic neuritis. J Exp Med. (2003) 197:1073–81. 10.1084/jem.20021603 12732654 PMC2193967

[13] Rodi M Dimisianos N de Lastic AL Sakellaraki P Deraos G Matsoukas J. Regulatory cell populations in relapsing-remitting multiple sclerosis (RRMS) patients: effect of disease activity and treatment regimens. Int J Mol Sci. (2016) 17:1398. 10.3390/ijms17091398 27571060 PMC5037678

[14] Rouse M Nagarkatti M Nagarkatti PS. The role of IL-2 in the activation and expansion of regulatory T-cells and the development of experimental autoimmune encephalomyelitis. Immunobiology. (2013) 218:674–82. 10.1016/j.imbio.2012.08.269 22954711 PMC3582788

[15] Haas J Hug A Viehover A Fritzsching B Falk CS Filser A. Reduced suppressive effect of CD4+CD25high regulatory T cells on the T cell immune response against myelin oligodendrocyte glycoprotein in patients with multiple sclerosis. Eur J Immunol. (2005) 35:3343–52. 10.1002/eji.200526065 16206232

[16] Danikowski KM Jayaraman S Prabhakar BS. Regulatory T cells in multiple sclerosis and myasthenia gravis. J Neuroinflamm. (2017) 14:117. 10.1186/s12974-017-0892-8 28599652 PMC5466736

[17] Vogel I Kasran A Cremer J Kim YJ Boon L Van Gool SW. CD28/CTLA-4/B7 costimulatory pathway blockade affects regulatory T-cell function in autoimmunity. Eur J Immunol. (2015) 45:1832–41. 10.1002/eji.201445190 25727069

[18] Zhou L Lopes JE Chong MM Ivanov II Min R Victora GD. TGF-beta-induced Foxp3 inhibits T(H)17 cell differentiation by antagonizing RORgammat function. Nature. (2008) 453:236–40. 10.1038/nature06878 18368049 PMC2597437

[19] Murai M Krause P Cheroutre H Kronenberg M. Regulatory T-cell stability and plasticity in mucosal and systemic immune systems. Mucosal Immunol. (2010) 3:443–9. 10.1038/mi.2010.27 20505662 PMC2924438

[20] Fourcade J Sun Z Chauvin JM Ka M Davar D Pagliano O. CD226 opposes TIGIT to disrupt tregs in melanoma. JCI Insight. (2018) 3:e121157. 10.1172/jci.insight.121157 30046006 PMC6124410

[21] Geng J Yu S Zhao H Sun X Li X Wang P The transcriptional coactivator TAZ regulates reciprocal differentiation of TH17 cells and treg cells. Nat Immunol. (2017) 18:800–12. 10.1038/ni.3748 28504697

[22] Pino PA Cardona AE. Isolation of brain and spinal cord mononuclear cells using percoll gradients. J Vis Exp. (2011) 48:2348. 10.3791/2348 21339713 PMC3339837

[23] Shibuya A Campbell D Hannum C Yssel H Franz-Bacon K McClanahan T. DNAM-1, a novel adhesion molecule involved in the cytolytic function of T lymphocytes. Immunity. (1996) 4:573–81. 10.1016/S1074-7613(00)70060-4 8673704

[24] Koyama M Kuns RD Olver SD Lineburg KE Lor M Teal BE. Promoting regulation via the inhibition of DNAM-1 after transplantation. Blood. (2013) 121:3511–20. 10.1182/blood-2012-07-444026 23430112

[25] Lozano E Dominguez-Villar M Kuchroo V Hafler DA. The TIGIT/CD226 axis regulates human T cell function. J Immunol. (2012) 188:3869–75. 10.4049/jimmunol.1103627 22427644 PMC3324669

[26] Quinn SM Cunningham K Raverdeau M Walsh RJ Curham L Malara A. Anti-inflammatory trained immunity mediated by helminth products attenuates the induction of T cell-mediated autoimmune disease. Front Immunol. (2019) 10:1109. 10.3389/fimmu.2019.01109 31178861 PMC6537856

[27] Zhang R Zeng H Zhang Y Chen K Zhang C Song C. CD226 ligation protects against EAE by promoting IL-10 expression via regulation of CD4+ T cell differentiation. Oncotarget. (2016) 7:19251–64. 10.18632/oncotarget.7834 26942885 PMC4991380

[28] Becher B Segal BM. T(H)17 cytokines in autoimmune neuro-inflammation. Curr Opin Immunol. (2011) 23:707–12. 10.1016/j.coi.2011.08.005 21907555 PMC3535446

[29] Rostami A Ciric B. Role of Th17 cells in the pathogenesis of CNS inflammatory demyelination. J Neurol Sci. (2013) 333:76–87. 10.1016/j.jns.2013.03.002 23578791 PMC3726569

[30] Zhang T Xu ZW Chen LH Zhang XH Wang DL Zhao ZW. Localization of CD226 in the mouse hippocampus and cerebellum during adulthood and postnatal development. Neuroscience. (2009) 158:766–75. 10.1016/j.neuroscience.2008.07.074 18793698

[31] Kim BS Lu H Ichiyama K Chen X Zhang YB Mistry NA. Generation of RORgammat^+^ antigen-specific T regulatory 17 cells from Foxp3^+^ precursors in autoimmunity. Cell Rep. (2017) 21:195–207. 10.1016/j.celrep.2017.09.021 28978473 PMC5716359

[32] Do J Kim D Kim S Valentin-Torres A Dvorina N Jang E. Treg-specific IL-27Ralpha deletion uncovers a key role for IL-27 in Treg function to control autoimmunity. Proc Natl Acad Sci USA. (2017) 114:10190–5. 10.1073/pnas.1703100114 28874534 PMC5617261

[33] Pandiyan P Zhu J. Origin and functions of pro-inflammatory cytokine producing Foxp3+ regulatory T cells. Cytokine. (2015) 76:13–24. 10.1016/j.cyto.2015.07.005 26165923 PMC4969074

[34] Zhao Q Cheng W Xi Y Cao Z Xu Y Wu T. IFN-beta regulates Th17 differentiation partly through the inhibition of osteopontin in experimental autoimmune encephalomyelitis. Mol Immunol. (2018) 93:20–30. 10.1016/j.molimm.2017.11.002 29127843

[35] Chaudhry A Samstein RM Treuting P Liang Y Pils MC Heinrich JM. Interleukin-10 signaling in regulatory T cells is required for suppression of Th17 cell-mediated inflammation. Immunity. (2011) 34:566–78. 10.1016/j.immuni.2011.03.018 21511185 PMC3088485

[36] Oh SA Liu M Nixon BG Kang D Toure A Bivona M. Foxp3-independent mechanism by which TGF-β controls peripheral T cell tolerance. Proc Natl Acad Sci USA. (2017) 114:E7536–44. 10.1073/pnas.1706356114 28827353 PMC5594672

[37] Lu L Wang J Zhang F Chai Y Brand D Wang X. Role of SMAD and non-SMAD signals in the development of Th17 and regulatory T cells. J Immunol. (2010) 184:4295–306. 10.4049/jimmunol.0903418 20304828 PMC3087811

[38] Shrestha S Yang K Guy C Vogel P Neale G Chi H. Treg cells require the phosphatase PTEN to restrain TH1 and TFH cell responses. Nat Immunol. (2015) 16:178–87. 10.1038/ni.3076 25559258 PMC4297581

[39] Hou S Ge K Zheng X Wei H Sun R Tian Z. CD226 protein is involved in immune synapse formation and triggers natural killer (NK) cell activation via its first extracellular domain. J Biol Chem. (2014) 289:6969–77. 10.1074/jbc.M113.498253 24451371 PMC3945358

[40] Xu Z Jin B. A novel interface consisting of homologous immunoglobulin superfamily members with multiple functions. Cell Mol Immunol. (2010) 7:11–9. 10.1038/cmi.2009.108 20081873 PMC4003259

[41] Lozano E Joller N Cao Y Kuchroo VK Hafler DA. The CD226/CD155 interaction regulates the proinflammatory (Th1/Th17)/anti-inflammatory (Th2) balance in humans. J Immunol. (2013) 191:3673–80. 10.4049/jimmunol.1300945 23980210 PMC3819731

[42] Sonar SA Lal G. Differentiation and transmigration of CD4 T cells in neuroinflammation and autoimmunity. Front Immunol. (2017) 8:1695. 10.3389/fimmu.2017.01695 29238350 PMC5712560

[43] Lee PW Severin ME Lovett-Racke AE. TGF-beta regulation of encephalitogenic and regulatory T cells in multiple sclerosis. Eur J Immunol. (2017) 47:446–53. 10.1002/eji.201646716 28102541 PMC5499671

[44] McClymont SA Putnam AL Lee MR Esensten JH Liu W Hulme MA. Plasticity of human regulatory T cells in healthy subjects and patients with type 1 diabetes. J Immunol. (2011) 186:3918–26. 10.4049/jimmunol.1003099 21368230 PMC3091943

[45] Wang YF Zhang Y Zhu Z Wang TY Morris DL Shen JJ. Identification of ST3AGL4, MFHAS1, CSNK2A2 and CD226 as loci associated with systemic lupus erythematosus (SLE) and evaluation of SLE genetics in drug repositioning. Ann Rheum Dis. (2018) 77:1078–84. 10.1136/annrheumdis-2018-213093 29625966

[46] Fuhrman CA Yeh WI Seay HR Saikumar Lakshmi P Chopra G Zhang L. Divergent phenotypes of human regulatory T cells expressing the receptors TIGIT and CD226. J Immunol. (2015) 195:145–55. 10.4049/jimmunol.1402381 25994968 PMC4475416

[47] Jamshidian A Shaygannejad V Pourazar A Zarkesh-Esfahani SH Gharagozloo M. Biased Treg/Th17 balance away from regulatory toward inflammatory phenotype in relapsed multiple sclerosis and its correlation with severity of symptoms. J Neuroimmunol. (2013) 262:106–12. 10.1016/j.jneuroim.2013.06.007 23845464

[48] Gao Q Zhang Y Han C Hu X Zhang H Xu X. Blockade of CD47 ameliorates autoimmune inflammation in CNS by suppressing IL-1-triggered infiltration of pathogenic Th17 cells. J Autoimmun. (2016) 69:74–85. 10.1016/j.jaut.2016.03.002 26994903

[49] Wang M Liu C Bond A Yang J Zhou X Wang J. Dysfunction of regulatory T cells in patients with ankylosing spondylitis is associated with a loss of Tim-3. Int Immunopharmacol. (2018) 59:53–60. 10.1016/j.intimp.2018.03.032 29625390

[50] Hull CM Peakman M Tree TIM. Regulatory T cell dysfunction in type 1 diabetes: what's broken and how can we fix it? Diabetologia. (2017) 60:1839–50. 10.1007/s00125-017-4377-1 28770318 PMC6448885

[51] Dardalhon V Schubart AS Reddy J Meyers JH Monney L Sabatos CA. CD226 is specifically expressed on the surface of Th1 cells and regulates their expansion and effector functions. J Immunol. (2005) 175:1558–65. 10.4049/jimmunol.175.3.1558 16034094

[52] Raphael I Nalawade S Eagar TN Forsthuber TG. T cell subsets and their signature cytokines in autoimmune and inflammatory diseases. Cytokine. (2015) 74:5–17. 10.1016/j.cyto.2014.09.011 25458968 PMC4416069

[53] Gu C Wu L Li X. IL-17 family: cytokines, receptors and signaling. Cytokine. (2013) 64: 477–85. 10.1016/j.cyto.2013.07.022 24011563 PMC3867811

[54] Hofmann SR Rosen-Wolff A Tsokos GC Hedrich CM. Biological properties and regulation of IL-10 related cytokines and their contribution to autoimmune disease and tissue injury. Clin Immunol. (2012) 143:116–27. 10.1016/j.clim.2012.02.005 22459704

[55] Yang XO Chang SH Park H Nurieva R Shah B Acero L. Regulation of inflammatory responses by IL-17F. J Exp Med. (2008) 205:1063–75. 10.1084/jem.20071978 18411338 PMC2373839

[56] Gaud G Roncagalli R Chaoui K Bernard I Familiades J Colacios C. The costimulatory molecule CD226 signals through VAV1 to amplify TCR signals and promote IL-17 production by CD4^+^ T cells. Sci Signal. (2018) 11:aar3083. 10.1126/scisignal 29991650

[57] Li X Zheng Y. Regulatory T cell identity: formation and maintenance. Trends Immunol. (2015) 36:344–53. 10.1016/j.it.2015.04.006 25981968 PMC4458194

[58] Sakaguchi S Vignali DA Rudensky AY Niec RE Waldmann H. The plasticity and stability of regulatory T cells. Nat Rev Immunol. (2013) 13:461–7. 10.1038/nri3464 23681097

[59] Cao J Zhang X Wang Q Wang X Jin J Zhu T. Cyclic AMP suppresses TGF-beta-mediated adaptive Tregs differentiation through inhibiting the activation of ERK and JNK. Cell Immunol. (2013) 285:42–8. 10.1016/j.cellimm.2013.08.006 24055734

[60] Pompura SL Dominguez-Villar M. The PI3K/AKT signaling pathway in regulatory T-cell development, stability, and function. J Leukoc Biol. (2018) 103:1065–76. 10.1002/jlb.2mir0817-349r 29357116

[61] Kumar P Marinelarena A Raghunathan D Ragothaman VK Saini S Bhattacharya P. Critical role of OX40 signaling in the TCR-independent phase of human and murine thymic Treg generation. Cell Mol Immunol. (2019) 16:138–53. 10.1038/cmi.2018.8 29578532 PMC6355936

[62] Lu L Ma J Wang X Wang J Zhang F Yu J. Synergistic effect of TGF-beta superfamily members on the induction of Foxp3+ treg. Eur J Immunol. (2010) 40:142–52. 10.1002/eji.200939618 19943263 PMC2837277

[63] Zhang Z Wu N Lu Y Davidson D Colonna M Veillette A. DNAM-1 controls NK cell activation via an ITT-like motif. J Exp Med. (2015) 212:2165–82. 10.1084/jem.20150792 26552706 PMC4647266

[64] Bryceson YT March ME Ljunggren HG Long EO. Synergy among receptors on resting NK cells for the activation of natural cytotoxicity and cytokine secretion. Blood. (2006) 107:159–66. 10.1182/blood-2005-04-1351 16150947 PMC1895346

[65] Wu T Mohan C. The AKT axis as a therapeutic target in autoimmune diseases. Endocr Metab Immune Disord Drug Targets. (2009) 9:145–50. 10.2174/187153009788452417 19519464

[66] Huang N Perl A. Metabolism as a target for modulation in autoimmune diseases. Trends Immunol. (2018) 39:562–76. 10.1016/j.it.2018.04.006 29739666

[67] Lisi L Navarra P Cirocchi R Sharp A Stigliano E Feinstein DL. Rapamycin reduces clinical signs and neuropathic pain in a chronic model of experimental autoimmune encephalomyelitis. J Neuroimmunol. (2012) 243:43–51. 10.1016/j.jneuroim.2011.12.018 22264993

[68] Hou H Miao J Cao R Han M Sun Y Liu X. Rapamycin ameliorates experimental autoimmune encephalomyelitis by suppressing the mTOR-STAT3 pathway. Neurochem Res. (2017) 42:2831–40. 10.1007/s11064-017-2296-7 28600752

[69] Delgoffe GM Pollizzi KN Waickman AT Heikamp E Meyers DJ Horton MR. The kinase mTOR regulates the differentiation of helper T cells through the selective activation of signaling by mTORC1 and mTORC2. Nat Immunol. (2011) 12:295–303. 10.1038/ni.2005 21358638 PMC3077821

[70] Fernandez D Bonilla E Mirza N Niland B Perl A. Rapamycin reduces disease activity and normalizes T cell activation-induced calcium fluxing in patients with systemic lupus erythematosus. Arthritis Rheum. (2006) 54:2983–8. 10.1002/art.22085 16947529 PMC4034146

[71] Keever-Taylor CA Browning MB Johnson BD Truitt RL Bredeson CN Behn B. Rapamycin enriches for CD4^+^ CD25^+^ CD27^+^ Foxp3^+^ regulatory T cells in *ex vivo*-expanded CD25-enriched products from healthy donors and patients with multiple sclerosis. Cytotherapy. (2007) 9:144–57. 10.1080/14653240601145223 17453966

[72] Chi H. Regulation and function of mTOR signalling in T cell fate decisions. Nat Rev Immunol. (2012) 12:325–38. 10.1038/nri3198 22517423 PMC3417069

[73] 17th International Congress of Immunology 19-23 October 2019 Beijing China Eur J Immunol. (2019) 49(Suppl. 3):1–2223. 10.1002/eji.201970400 PMC7163613 31592543

