# Supplementary figures and images for: CD226 Attenuates Treg Proliferation via Akt and Erk Signaling in an EAE Model
Source: Front Immunol. 2020 Aug 21;11:1883. doi: 10.3389/fimmu.2020.01883 (PMC7478170; doi:10.3389/fimmu.2020.01883)

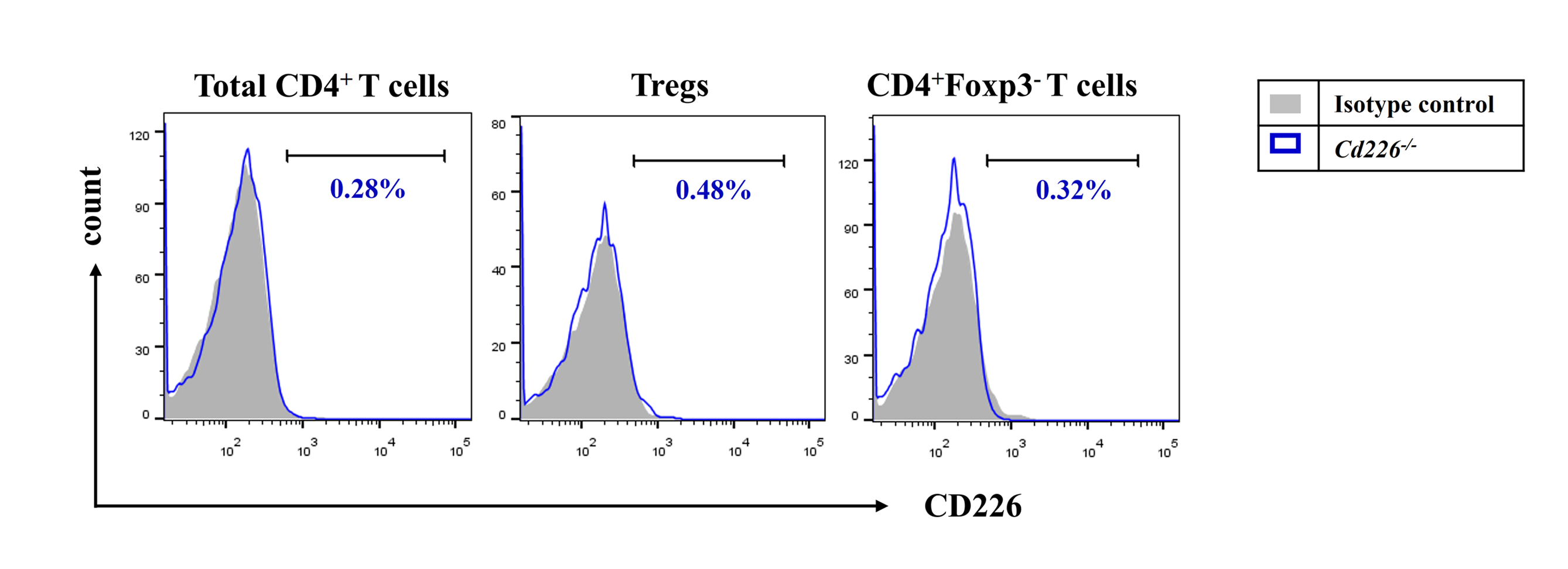

Supplement: Supplemental Figure 1 — Efficient CD226 deletion in Cd226−/− mice. Total CD4+ T cells, Tregs (CD4+ CD25+ Foxp3+) and Tconv cells (CD4+ Foxp3−) were gated from the splenocytes of Cd226−/− mice, and the expression levels of CD226 were detected by FCM (n = 8). The data are representative of four independent experiments. The numbers in each quadrant show the percentage of the relevant cell population. [file Image_1.TIF]

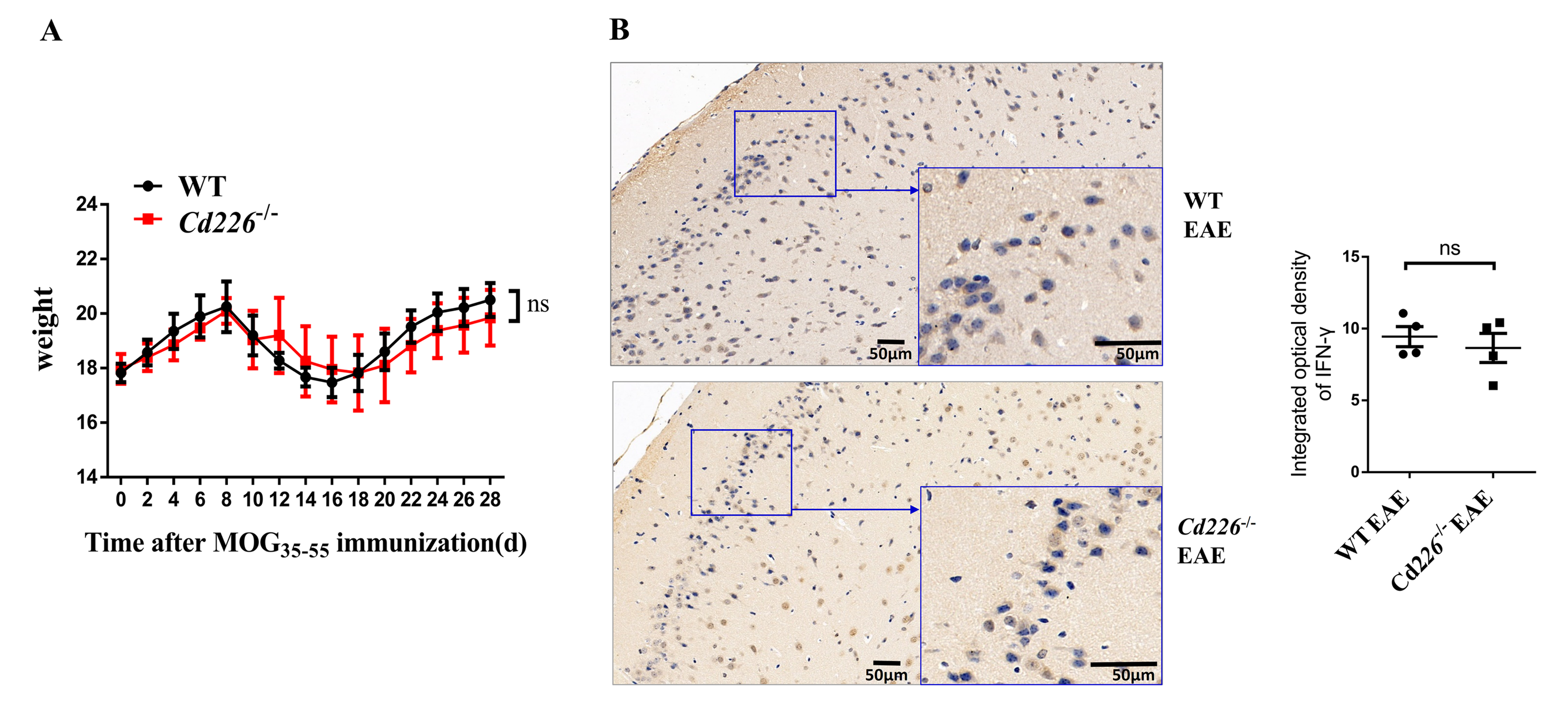

Supplement: Supplemental Figure 2 — Characterization of WT EAE and Cd226−/− EAE mice. (A) The body weight of Cd226−/− and WT mice was monitored daily after immunization with MOG35−55 (n = 16). The results represent eight independent experiments. (B) IFN-γ expression in the brain on days 15–18 post-immunization was detected by immunohistochemical staining (n = 6), and the integrated optical density of IFN-γ was measured with ImageJ. The results represent three independent experiments. nsP > 0.05. [file Image_2.TIF]

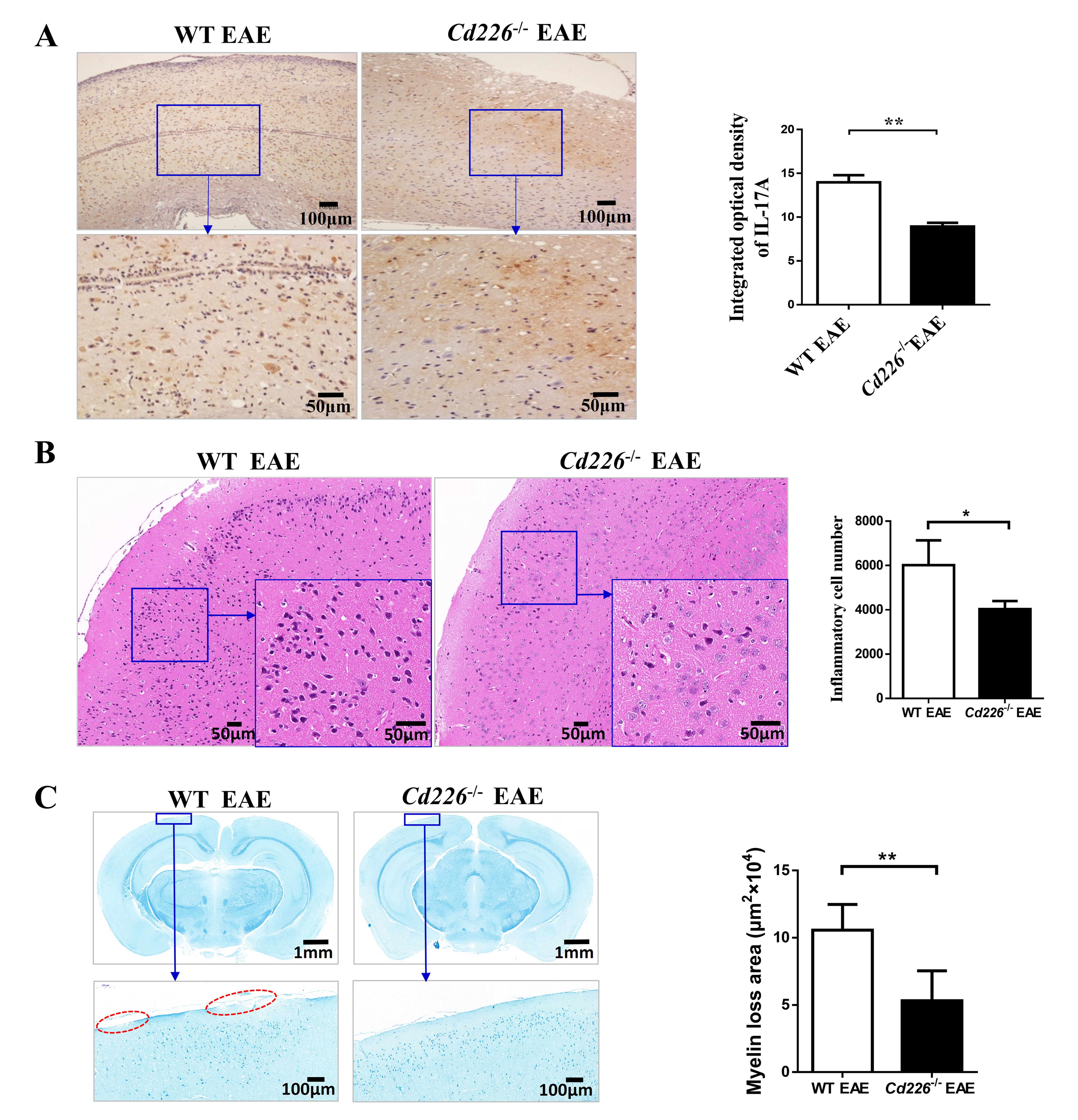

Supplement: Supplemental Figure 3 — Lower levels of infiltrated cells and demyelination in the CNS of Cd226−/− Mice. (A) IL-17A expression in the lumbar region of the spinal cord (longitudinal sections) at days 15–18 post-immunization was detected by immunohistochemical staining (n = 6) (scale bars indicate 100 and 50 μm for lower and higher magnification, respectively). (B) Histological analysis of lymphocyte infiltration in the brain tissue (cross-sections) of the indicated mice by H&E staining (scale bars indicate 50 μm for lower magnification, and 50 μm for higher magnification) (n = 6). (C) LFB staining of the brains (cross-sections) of WT and Cd226−/− mice at the peak of EAE (n = 6). Demyelination of representative brain sections is indicated by loss of blue staining (scale bars indicate 1 mm and 100 μm for lower and higher magnification, respectively), and the total demyelinated area was measured with ImageJ. The results represent at least two independent experiments (A–C). *P < 0.05, **P < 0.005. [file Image_3.TIF]

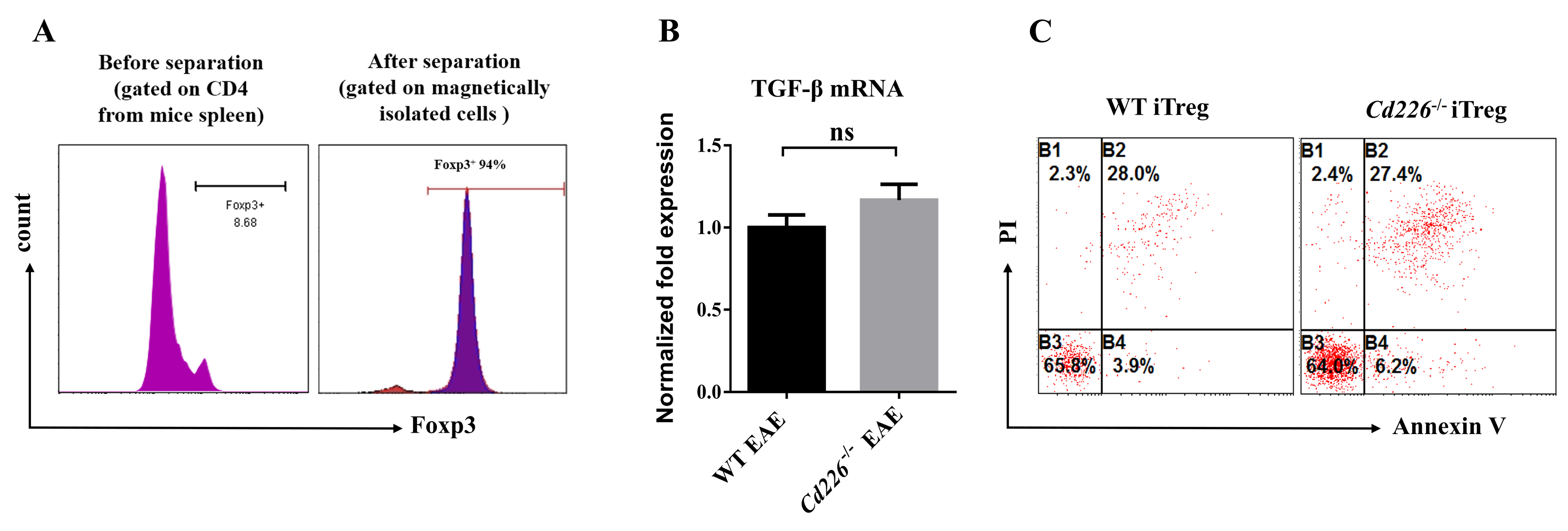

Supplement: Supplemental Figure 4 — Cd226−/− iTregs do not display decreased apoptosis. (A) The purity of Tregs before and after magnetic separation was determined by FCM (n = 6). (B) The relative mRNA expression levels of TGF-β in splenic Tregs from WT or Cd226−/− mice during EAE were assessed by qRT-PCR. (C) Annexin V and PI staining of iTregs after polarization from WT and Cd226−/− naïve CD4+ T cells for 3 days (n = 6). The results represent two or three independent experiments (A–C). The numbers in each quadrant show the percentage of the relevant cell population. [file Image_4.TIF]
