# Supplementary material for: CD226 Attenuates Treg Proliferation via Akt and Erk Signaling in an EAE Model
Source: Front Immunol. 2020 Aug 21;11:1883. doi: 10.3389/fimmu.2020.01883 (PMC7478170; doi:10.3389/fimmu.2020.01883)
Supplement: Supplemental Table 1 — The primers used for qRT-PCR in this study are listed. [file Table_1.DOCX]

| IFN-γ | 5’ ATGAACGCTACACACTGCATC 3’ |
| --- | --- |
|  | 5’ CCATCCTTTTGCCAGTTCCTC 3’ |
| IL-4 | 5’ GGTCTCAACCCCCAGCTAGT 3’ |
|  | 5’ GCCGATGATCTCTCTCAAGTGAT 3’ |
| IL-17A | 5’ TTTAACTCCCTTGGCGCAAAA 3’ |
|  | 5’ CTTTCCCTCCGCATTGACAC 3’ |
| IL-10 | 5’ CTTACTGACTGGCATGAGGATCA 3’ |
|  | 5’ GCAGCTCTAGGAGCATGTGG 3’ |
| Foxp3 | 5’ CCCATCCCCAGGAGTCTTG 3’ |
|  | 5’ ACCATGACTAGGGGCACTGTA 3’ |
| RORγt | 5’ GACCCACACCTCACAAATTGA 3’ |
|  | 5’ AGTAGGCCACATTACACTGCT 3’ |
| TGF-β | 5’ CTCCCGTGGCTTCTAGTGC 3’ |
|  | 5’ GCCTTAGTTTGGACAGGATCTG 3’ |
| GAPDH | 5’ AGGTCGGTGTGAACGGATTTG 3’ |
|  | 5’ TGTAGACCATGTAGTTGAGGTCA 3’ |

Supplemental Table 1. The primers used for qRT-PCR in this study are listed.
